# A combined transcriptomic and physiological approach to understanding the adaptive mechanisms to cope with oxidative stress in *Fusarium graminearum*

**DOI:** 10.1128/spectrum.01485-23

**Published:** 2023-09-06

**Authors:** Jiyeun Park, Hyun-Hee Lee, Heeji Moon, Nahyun Lee, Sieun Kim, Jung-Eun Kim, Yoonji Lee, Kyunghun Min, Hun Kim, Gyung Ja Choi, Yin-Won Lee, Young-Su Seo, Hokyoung Son

**Affiliations:** 1 Department of Agricultural Biotechnology, Seoul National University, Seoul, Republic of Korea; 2 Department of Integrated Biological Science, Pusan National University, Busan, Republic of Korea; 3 Research Institute of Climate Change and Agriculture, National Institute of Horticultural and Herbal Science, Jeju, Republic of Korea; 4 Center for Eco-friendly New Materials, Korea Research Institute of Chemical Technology, Daejeon, Republic of Korea; 5 Research Institute of Agriculture and Life Sciences, Seoul National University, Seoul, Republic of Korea; Universita degli Studi del Molise, Campobasso, Italy

**Keywords:** oxidative stress response, *Fusarium graminearum*, DNA damage response, autophagy, ubiquitin-proteasome pathway, heme biosynthesis

## Abstract

In plant-pathogen interactions, oxidative bursts are crucial for plants to defend themselves against pathogen infections. Rapid production and accumulation of reactive oxygen species kill pathogens directly and cause local cell death, preventing pathogens from spreading to adjacent cells. Meanwhile, the pathogens have developed several mechanisms to tolerate oxidative stress and successfully colonize plant tissues. In this study, we investigated the mechanisms responsible for resistance to oxidative stress by analyzing the transcriptomes of six oxidative stress-sensitive strains of the plant pathogenic fungus *Fusarium graminearum*. Weighted gene co-expression network analysis identified several pathways related to oxidative stress responses, including the DNA repair system, autophagy, and ubiquitin-mediated proteolysis. We also identified hub genes with high intramodular connectivity in key modules and generated deletion or conditional suppression mutants. Phenotypic characterization of those mutants showed that the deletion of *FgHGG4*, *FgHGG10*, and *FgHGG13* caused sensitivity to oxidative stress, and further investigation on those genes revealed that transcriptional elongation and DNA damage responses play roles in oxidative stress response and pathogenicity. The suppression of *FgHGL7* also led to hypersensitivity to oxidative stress, and we demonstrated that *FgHGL7* plays a crucial role in heme biosynthesis and is essential for peroxidase activity. This study increases the understanding of the adaptive mechanisms to cope with oxidative stress in plant pathogenic fungi.

**IMPORTANCE:**

Fungal pathogens have evolved various mechanisms to overcome host-derived stresses for successful infection. Oxidative stress is a representative defense system induced by the host plant, and fungi have complex response systems to cope with it. *Fusarium graminearum* is one of the devastating plant pathogenic fungi, and understanding its pathosystem is crucial for disease control. In this study, we investigated adaptive mechanisms for coping with oxidative stress at the transcriptome level using oxidative stress-sensitive strains. In addition, by introducing genetic modification technique such as CRISPR-Cas9 and the conditional gene expression system, we identified pathways/genes required for resistance to oxidative stress and also for virulence. Overall, this study advances the understanding of the oxidative stress response and related mechanisms in plant pathogenic fungi.

## INTRODUCTION

Reactive oxygen species (ROS) are by-products generated during aerobic metabolism (1, 2). In general, ROS exist as superoxide radicals, hydrogen peroxide (H_2_O_2_), and hydroxyl radicals, and they function as signaling molecules through the oxidation of signal proteins or by themselves in the cell (3, 4). However, when cellular ROS accumulate to a high level, they can cause protein oxidation, lipid peroxidation, and/or DNA damage, all of which can lead to cell death (5 – 7).

Plants have evolved redox-related signals to deal with various biotic and abiotic stressors (8, 9). In plant-pathogen interactions, plants utilize ROS as signal molecules to regulate immune responses and physiologically inhibit pathogen infection. Plants recognize pathogen-associated molecular patterns or damage-associated molecular patterns through pattern recognition receptors in the cell membrane that then trigger pattern-triggered immunity (PTI) (10, 11). During PTI, rapid production of H_2_O_2_ and superoxide anions is concentrated in plant cells via NADPH-oxidase, peroxidases, and other ROS sources in a process generally known as an oxidative burst (12 – 14). An oxidative burst causing local cell death at the infection site can kill pathogens directly and inhibit the invasion of pathogens to the adjacent cell (15, 16).

Pathogens, meanwhile, have evolved several mechanisms to overcome oxidative bursts in host plants. ROS scavenging mechanisms are generally divided into enzymatic and non-enzymatic systems, and those two systems respond in a coordinated manner to oxidative stress (17, 18). Peroxidase, catalase, and superoxide dismutase (SOD) are representative enzymatic antioxidants, and glutathione and ascorbic acid are non-enzymatic antioxidants. These ROS-detoxifying systems have been studied thoroughly in plant pathogenic fungi. For example, peroxidases have been identified, and their roles in oxidative stress resistance have been characterized in *Fusarium graminearum* and *Magnaporthe oryzae* (19, 20). Functional analyses have revealed the role of SOD in oxidative stress resistance and virulence in *Sclerotinia sclerotiorum*, *Botrytis cinerea*, and *Verticillium dahliae* (21 – 23). The transcription factors such as Ap1, Skn7, and Atf1 regulating these antioxidant systems have also been extensively investigated, and it has been confirmed that their role in oxidative stress response is highly conserved in plant pathogenic fungi (24 – 26).

In addition to these antioxidant systems, mechanisms for recovering oxidative damage are essential for survival under oxidative stress conditions. DNA damage and protein oxidation are representative oxidative damage. DNA damage responses that identify and repair damaged DNA have been investigated in plant pathogenic fungi, including *Ustilago maydis*, *V. dahliae*, and *F. graminearum* (27 – 30). Also, proteolysis system to eliminate the damaged protein, including the ubiquitin-proteasome system (UPS), has been studied in *M. oryzae* and *Fusarium oxysporum* (31 – 33). However, a genome-wide correlation between these mechanisms and oxidative stress is required, and further identification of the mechanisms that contribute to coping with oxidative stress is still needed.

*F. graminearum* is one of the most destructive plant pathogenic fungi, causing Fusarium head blight (FHB) in important cereal crops worldwide (34, 35). Infection of *F. graminearum,* which can lead to severe yield losses and the accumulation of mycotoxins on grains, is an important cause of food shortages and a serious threat to public health (36). To control FHB, it is essential to understand how the fungus overcomes plant defense mechanisms and invades host plant cell. In this study, we aimed to identify the mechanisms/genes involved in responding to oxidative stress through a combined transcriptomic and physiological approach, and to explore the role of these mechanisms in the oxidative stress response and pathogenicity in *F. graminearum*.

## RESULTS AND DISCUSSION

### Weighted gene co-expression network analysis and identification of co-expression modules

**Fig 1:**
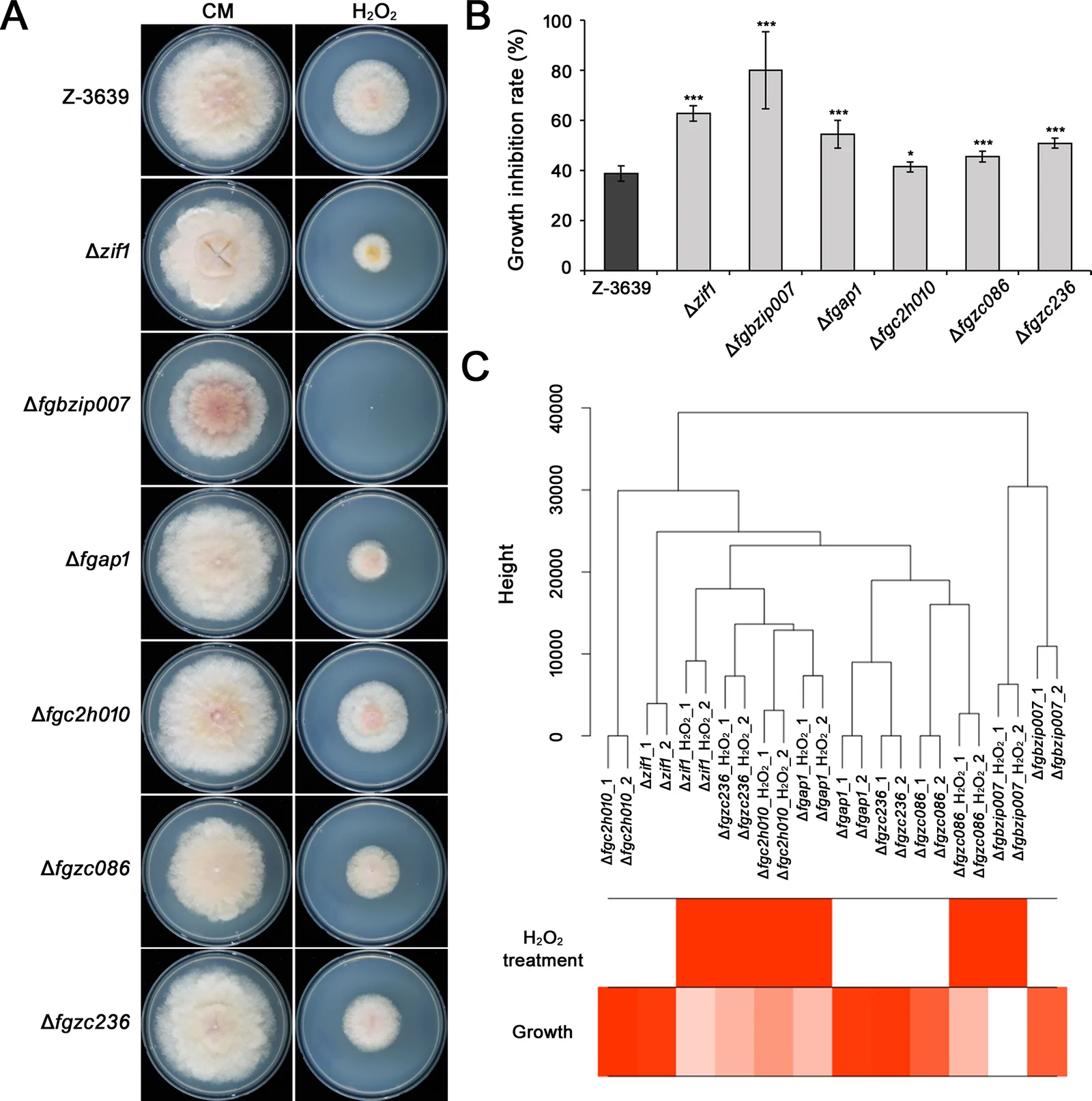
Phenotypic and transcriptome analyses of oxidative stress-sensitive strains. (**A**) Mycelial growth of six oxidative stress-sensitive mutants under oxidative stress. Each strain was inoculated on CM and CM supplemented with 10 mM H_2_O_2_. Photographs were taken 5 days after inoculation. (**B**) Statistical analysis of mycelial growth inhibition under oxidative stress. Error bars represent standard deviations. Asterisks represent significant differences from the wild type (*P < 0.05; ***P < 0.001; *t*-test). (**C**) A clustering dendrogram of samples with trait heatmap. The 24 samples were clustered based on mRNA expression data. The color band underneath the tree indicates the value of treated H_2_O_2_ concentration and colony diameter. Linear gradation colors from white to red represent the values of each trait corresponding to the sample.

We aimed to identify the oxidative stress response or oxidative stress-related mechanisms based on a comprehensive transcriptome analysis. We hypothesized that the expression of core genes involved in oxidative stress resistance would be dramatically changed in stress-sensitive strains than that of the wild type. In *F. graminearum*, eight transcription factor mutants displayed hypersensitivity to oxidative stress (20). We cultured these mutants on complete media (CM) with or without 10 mM H_2_O_2_ supplementation, and six mutants exhibiting dramatic growth defects compared with the wild-type strain were selected for further RNA-seq analyses: Δ*zif1*, Δ*fgc2h010,* Δ*fgzc236*, Δ*fgzc086*, Δ*fgap1*, and Δ*fgbzip007* (Fig. 1A). The inhibition rates of Δ*zif1* and Δ*fgbzip007* were approximately twofold higher than that of the wild-type strain, and other strains showed significantly altered growth under oxidative stress conditions compared with the wild type (Fig. 1B). These six mutants were defined as “sensitive strains” in this study.

To reveal the mechanism related to oxidative stress response, we analyzed the transcriptomes of the six strains using RNA-seq data and conducted a weighted gene co-expression network analysis (WGCNA). All 24 samples were clustered, and samples derived from strains treated with H_2_O_2_ were clustered together, except those of Δ*fgbzip007* and Δ*fgzc086* (Fig. 1C). This clustering suggests that changes in gene expression for each sample are affected more by oxidative stress than by differences among genotypes.

**Fig 2:**
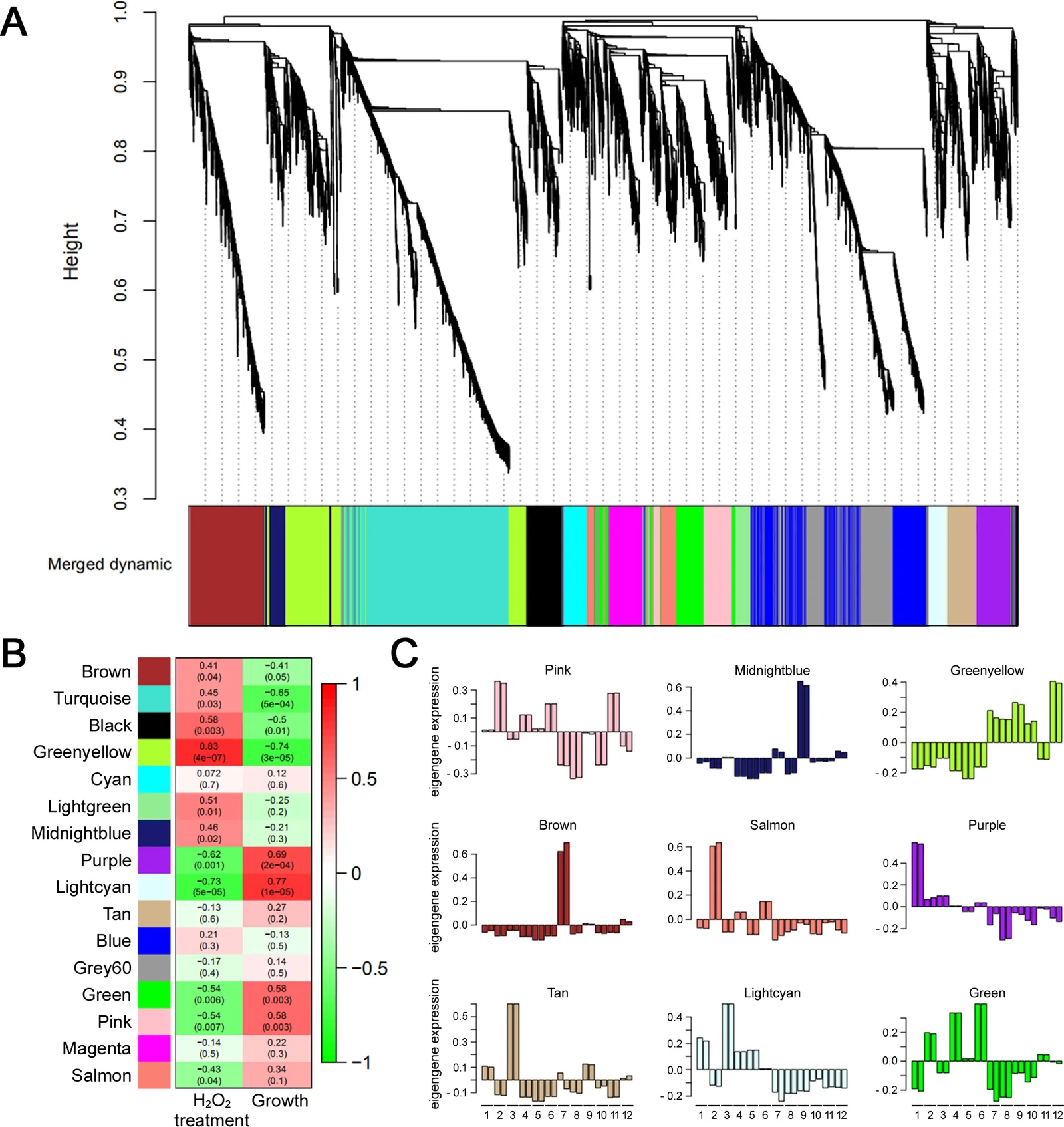
Weighted gene co-expression network analysis. (**A**) Clustering dendrogram of genes. The genes were clustered based on the dissimilarity of the topological overlap. (**B**) Module-trait relationships. Each cell contains the corresponding correlation value and *P*-value. (**C**) Eigengene expression patterns in nine modules. 1-Δ*zif1*, 2-Δ*fgbzip007*, 3-Δ*fgc2h010*, 4-Δ*fgzc236*, 5-Δ*fgzc086*, 6-Δ*fgap1*, 7-Δ*zif1*_H_2_O_2_, 8-Δ*fgzip007*_H_2_O_2_, 9-Δ*fgc2h010*_H_2_O_2_, 10-Δ*fgzc236*_H_2_O_2_, 11-Δ*fgzc086*_H_2_O_2_, 12-Δ*fgap1*_H_2_O_2_.

Radial growth of mutants and H_2_O_2_ treatment were used as phenotypic traits for WGCNA analysis, and total gene profiles were divided into 16 modules based on transcription patterns (Fig. 2A). Among the 16 modules, three were closely correlated with H_2_O_2_ treatment and growth rate (*P* ≤ 0.001): Greenyellow, Lightcyan, and Purple (Fig. 2B). Gene expression patterns of the Greenyellow module were positively correlated with H_2_O_2_ treatment and negatively correlated with growth rate, which indicates that the Greenyellow module includes genes that increase expression when radial growth of the strains decreases due to oxidative stress. Conversely, the gene expression patterns of the Lightcyan and Purple modules were negatively correlated with H_2_O_2_ treatment and positively correlated with growth. The Lightcyan and Purple modules include genes for which expression decreases when radial growth of the strain decreases due to oxidative stress. The eigengene expression in each sample indicated that the Greenyellow, Lightcyan, and Purple modules were closely correlated with oxidative stress (Fig. 2C). These results imply that genes of each module can be functionally related and co-regulated by H_2_O_2_ treatment because genes with strongly correlated expression levels tend to share similar biological functions (37).

### Functional enrichment analysis of genes in the high-correlation modules

To explore the biological process of genes included in each module, Kyoto Encyclopedia of Genes and Genomes (KEGG) enrichment analysis was performed for the three modules (Table 1). Among those genes, 245, 46, and 121 in the Greenyellow, Lightcyan, and Purple modules, respectively, had known functions. Genes in the Greenyellow module were mainly enriched in the “biosynthesis of amino acid pathway” (fgr01230), followed by “nucleotide excision repair” (fgr03420), “RNA transport” (fgr03013), and “protein processing in endoplasmic reticulum” (fgr04141). In the Lightcyan module, gene functions were enriched in the “metabolic pathway” (fgr01100) and “biosynthesis of secondary metabolites” (fgr01110). In the Purple module, genes were enriched in “metabolic pathways” (fgr01100), “oxidative phosphorylation” (fgr00190), the “MAPK signaling pathway” (fgr04011), and “Tricarboxylic acid cycle” (fgr00020) (Table 1).

**TABLE 1:** KEGG pathway enrichment analysis of genes in co-expression modules

Module	KEGG ID	Pathway	Count	*P*-value
Greenyellow	fgr03420	Nucleotide excision repair	16	2.75E−07
	fgr03440	Homologous recombination	9	2.49E−06
	fgr03450	Non-homologous end-joining	5	4.13E−04
	fgr03022	Basal transcription factors	10	7.21E−04
	fgr00400	Phenylalanine, tyrosine, and tryptophan biosynthesis	9	7.90E−04
	fgr03410	Base excision repair	8	8.08E−04
	fgr01230	Biosynthesis of amino acids	25	1.04E−03
	fgr04141	Protein processing in endoplasmic reticulum	15	9.63E−03
	fgr03013	RNA transport	16	1.04E−02
	fgr03430	Mismatch repair	6	1.39E−02
	fgr02010	ABC transporters	8	1.59E−02
	fgr04136	Autophagy	6	1.75E−02
	fgr03050	Proteasome	8	1.89E−02
	fgr04120	Ubiquitin-mediated proteolysis	10	3.33E−02
	fgr04138	Autophagy	12	3.36E−02
Lightcyan	fgr01100	Metabolic pathways	39	4.95E−07
	fgr00260	Glycine, serine, and threonine metabolism	5	6.17E−03
	fgr00600	Sphingolipid metabolism	3	1.31E−02
	fgr00430	Taurine and hypotaurine metabolism	2	1.70E−02
	fgr00100	Steroid biosynthesis	3	1.75E−02
	fgr00230	Purine metabolism	4	2.31E−02
	fgr01110	Biosynthesis of secondary metabolites	15	2.71E−02
	fgr00250	Alanine, aspartate, and glutamate metabolism	3	4.50E−02
Purple	fgr00190	Oxidative phosphorylation	21	1.21E−09
	fgr04011	MAPK^*a*^ signaling pathway	13	9.82E−06
	fgr01100	Metabolic pathways	82	2.16E−05
	fgr00020	Tricarboxylic acid cycle	7	8.67E−04
	fgr01040	Biosynthesis of unsaturated fatty acids	4	2.84E−03
	fgr01212	Fatty acid metabolism	7	5.63E−03
	fgr04139	Mitophagy	5	2.09E−02
	fgr04933	AGE-RAGE signaling pathway in diabetic complications	3	2.85E−02
	fgr04113	Meiosis	7	4.42E−02

^*a*^ MAPK: mitogen activated protein kinase

Gene ontology (GO) enrichment analysis was also performed on the three gene modules (Table S1). In the biological process category, genes in the Greenyellow module were enriched in “regulation of transcription, DNA-templated” (GO:0006355), “DNA repair” (GO:0006281), “DNA recombination” (GO:0006310), and “ubiquitin-dependent protein catabolic process” (GO:0006511). In the Lightcyan and Purple modules, genes were mainly enriched in the “oxidation-reduction process” (GO:0055114) and “protein phosphorylation” (GO:0006468), respectively.

KEGG and GO functional enrichment analysis revealed that DNA damage response and protein degradation mechanisms were notably enriched in the gene group of the Greenyellow module. These mechanisms have been reported to having essential roles for protecting the cell against ROS-derived damage. DNA damage response is an important cellular process in the repair of oxidative damage in DNA (38, 39). Autophagy and the ubiquitin-proteasome system are the primary intracellular protein degradation mechanisms and play a role in the removal of proteins damaged by oxidative stress (40 – 42). Collectively, these results indicate that the DNA damage repair system, autophagy, and ubiquitin-proteasome system are upregulated to repair the damage caused by oxidative stress in *F. graminearum*. Furthermore, the identification of these mechanisms suggests that a transcriptomic analysis approach using stress-sensitive strains is effective for further investigating adaptive mechanisms against oxidative stress.

### Identification of hub genes and construction of hub gene deletion mutants

We identified the top 30 genes with high intramodular connectivity as potential “hub” genes in each module to obtain insight into the regulatory response to oxidative stress. Among the hub genes, 16 had a KEGG orthology annotation in the Greenyellow module, and seven and eight genes were included in the Lightcyan and Purple modules, respectively (Table 2). We then constructed knock-out mutants for each gene to elucidate which genes are involved in oxidative stress response. We conducted fungal transformation using a homologous recombination method and confirmed gene deletions with diagnostic polymerase chain reaction (PCR) amplification followed by Southern blot hybridization (Fig. S3). Among the 31 hub genes, 26 were successfully deleted using a conventional fungal transformation method.

**TABLE 2:** Hub genes with KEGG annotation in each module

Module	Gene name	Locus ID	KO^*a*^ identifier	Definition	Reference
Greenyellow	*FgHGG1*	FGSG_01035	K03515	DNA repair protein REV1	This study
	*FgHGG2*	FGSG_01132	K08991	Crossover junction endonuclease MUS81	This study
	*FgHGG3*	FGSG_01932	K01760	Cysteine-S-conjugate beta-lyase	This study
	*FgHGG4*	FGSG_04274	K11375	Elongator complex protein 4	This study
	*FgHGG5*	FGSG_05904	K15083	DNA repair protein RAD16	This study
	*FgHGG6*	FGSG_05905	K15082	DNA repair protein RAD7	This study
	*FgHGG7*	FGSG_06098	K01940	Argininosuccinate synthase	This study
	*FgHGG8*	FGSG_06510	K08329	Autophagy-related protein 17	This study
	*GzNF002*	FGSG_07076	K12236	Transcriptional repressor NF-X1	(43)
	*FgHGG10*	FGSG_07962	K10875	DNA repair and recombination protein RAD54 and RAD54-like protein	This study
	*FgHGG11*	FGSG_08405	K03657	DNA helicase II/ATP-dependent DNA helicase PcrA	This study
	*FgHGG12*	FGSG_08785	K13431	Signal recognition particle receptor subunit alpha	This study
	*FgHGG13*	FGSG_10158	K10873	DNA repair and recombination protein RAD52	This study
	*FgHGG14*	FGSG_10352	K05864	Peptidyl-prolyl isomerase D	This study
	*FgHGG15*	FGSG_11781	K14284	Nuclear RNA export factor	This study
	*FgHGG16*	FGSG_12711	K10884	ATP-dependent DNA helicase 2 subunit 1	This study
Lightcyan	*FgHGL1*	FGSG_01672	K18278	Pyrimidine precursor biosynthesis enzyme	This study
	*FgERG3A*	FGSG_02502	K00227	Delta7-sterol 5-desaturase	(44)
	*FgHGL3*	FGSG_02978	K08139	MFS transporter, SP family, sugar:H + symporter	This study
	*FgHGL4*	FGSG_04458	K05916	Nitric oxide dioxygenase	This study
	*FgHGL5*	FGSG_09373	K18561	FAD-dependent fumarate reductase	This study
	*FgHGL6*	FGSG_09845	K13076	Sphingolipid 8-(E)-desaturase	This study
	*FgHGL7*	FGSG_10739	K00228	Coproporphyrinogen III oxidase	This study
Purple	*FgHGP1*	FGSG_00866	K15728	Phosphatidate phosphatase LPIN	This study
	*FgHGP2*	FGSG_03816	K01053	Gluconolactonase	This study
	*FgHGP3*	FGSG_05321	K00667	Fatty acid synthase subunit alpha, fungi type	This study
	*FgHGP4*	FGSG_05322	K00668	Fatty acid synthase subunit beta, fungi type	This study
	Δ*12-desaturase*	FGSG_05784	K10256	Omega-6 fatty acid desaturase/acyl-lipid omega-6 desaturase (delta-12 desaturase)	(45)
	*FgHGP6*	FGSG_06736	K17775	Mitochondrial distribution and morphology protein 34	This study
	*GzC2H050*	FGSG_07310	K11215	Transcription factor STE12	(43)
	*FgHGP8*	FGSG_09012	K00236	Succinate dehydrogenase (ubiquinone) cytochrome b560 subunit	This study

^*a*^ KO: KEGG orthology

**Fig 3:**
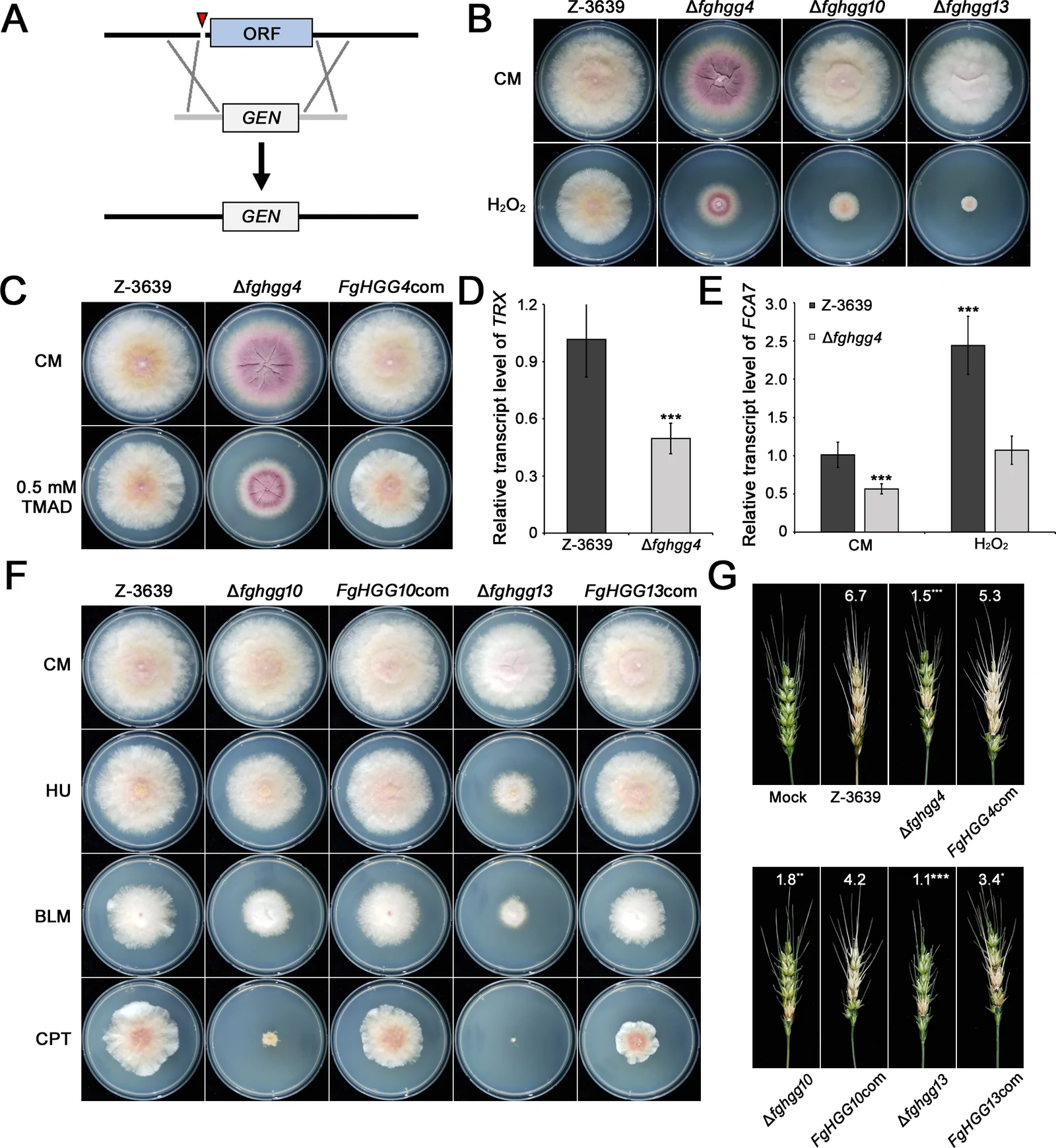
Redox homeostasis and the DNA repair system are important for oxidative stress resistance. (**A**) An overview of the strategy for the fungal transformation using the CRISPR/Cas9 system. The red triangle indicates the cleavage site induced by the Cas9 protein. *GEN*, geneticin resistance gene cassette. (**B**) Vegetative growth of hub gene deletion mutants under oxidative stress conditions. Each strain was cultured on CM and CM supplemented with 10 mM H_2_O_2_. Photographs were taken 5 days after inoculation. (**C**) Vegetative growth of the fungal strains on CM with or without diamide (0.5 mM). Photographs were taken 5 days after inoculation. (**D**) Relative transcript levels of thioredoxin (*TRX*) in the wild-type and Δ*fghgg4* deletion strains. The transcript levels of the gene were analyzed by quantitative real-time PCR amplification (qRT-PCR), and *CYP1* was used as a housekeeping gene. Asterisks represent significant differences from the wild type (***P < 0.001; *t*-test). (**E**) Transcript levels of *FCA7* in the wild-type and Δ*fghgg4* deletion strains. Total RNA was extracted from each strain grown for 30 min in CM and CM supplemented with 5 mM H_2_O_2_. The transcripts of *FCA7* were analyzed by qRT-PCR, and the *CYP1* was used as a housekeeping gene (***P < 0.001; *t*-test). (**F**) Sensitivity to DNA-damaging agents. Pictures were taken 5 days after inoculation on CM and CM treated with various DNA-damaging agents (10 mM hydroxyurea, HU; 10 µg/mL bleomycin, BLM; 0.2 mM camptothecin, CPT). (**G**) Virulence of the wild-type and hub gene mutant strains. Pictures were taken 2 weeks after inoculation. The average of infected spikes was used as a disease index and denoted with white letter. Asterisks represent significant differences from the wild type (*P < 0.05; **P < 0.01; ***P < 0.001; *t*-test).

We failed to delete *FgHGG4*, *FgHGG12*, *FgHGL7*, *FgHGP3*, and *FgHGP6* in three independent attempts. Among those genes, *FgHGG12* and *FgHGL7* are orthologs of the *Saccharomyces cerevisiae* genes *SRP101* and *HEM13*, respectively, which are essential in yeast (46). Except for those essential genes, we introduced CRISPR/Cas9 system to fungal transformation for the deletion of the genes. CRISPR/Cas9, a genome-editing technology derived from a bacterial adaptive immune system, enables targeted precise editing (47, 48). Several studies have reported that the genomic nick induced by Cas9 increases homologous recombination repair efficiency (49 – 51), and CRISPR/Cas9 has been applied previously to *Fusarium* species (52 – 54). To increase gene deletion efficiency for *FgHGG4*, *FgHGP3,* and *FgHGP6*, fungal transformation was conducted with preassembled Cas9 ribonucleoproteins, and the nick site was near the start codon (Fig. 3A). Finally, we successfully obtained Δ*fghgg4* deletion mutants. A total of 27 hub gene mutants were cultured under oxidative stress conditions, and three mutants, Δ*fghgg4*, Δ*fghgg10*, and Δ*fghgg13*, showed increased sensitivity to oxidative stress compared with the wild-type strain (Fig. 3B).

### The role of *FgHGG4*, *FgHGG10,* and *FgHGG13* in oxidative stress response

*FgHGG4* is predicted to be an ortholog of *ELP4* in *S. cerevisiae*, which is a subunit of the elongator complex protein. The elongator complex is composed of six subunits, named Elongator complex proteins 1 to 6 ( ELP1-6), and a holo-elongator is involved in transcriptional elongation by associating with an RNA polymerase II holoenzyme (55). In *Schizosaccharomyces pombe*, the elongator complex is important for oxidative stress resistance by altering the translation level of oxidative stress-related transcription factor Atf1 and Pcr1 via tRNA modification (56). The ELP complex is also known to be involved in the elongation of genes combating thiol oxidation in *S. cerevisiae* (57). To determine the role of FgHgg4 proteins in redox regulatory systems of *F. graminearum*, Δ*fghgg4* deletion mutants were cultured on CM supplemented with a thiol-specific oxidant, diamide. The deletion mutant exhibited growth defects compared with the wild-type and complementation strains (Fig. 3C). Transcription of the thioredoxin gene (*TRX*) was decreased by approximately 50% in Δ*fghgg4* deletion mutants compared with the wild type (Fig. 3D). A previous study confirmed that Elp3, one of the core subunits of the elongator complex, plays an important role in oxidative stress response in *F. graminearum* (58). In *elp3* deletion mutants, several peroxidases genes, including *FCA7*, were downregulated or unchanged compared with those of the wild-type strain under oxidative stress conditions. We determined the transcript levels of *FCA7*, the major peroxidase in *F. graminearum*, and quantitative real-time (qRT)-PCR results indicated that *FCA7* was downregulated in Δ*fghgg4* deletion mutants compared with the wild-type strain (Fig. 3E). The transcript level of *FCA7* in Δ*fghgg4* deletion mutants was increased under oxidative stress conditions, but was still 2.5-fold lower than that of the wild-type strain. These results indicate that the elongator complex is involved in redox homeostasis and peroxidase regulation, and deletion of its subunit caused increased sensitivity to oxidative stress.

*FgHGG10* and *FgHGG13* are the respective ortholog genes of *S. cerevisiae RAD54* and *RAD52*, which are involved in the repair of DNA double-strand breaks (59, 60). To confirm the role of *FgHGG10* and *FgHGG13* in DNA repair in *F. graminearum*, deletion mutants were cultured on media supplemented with various DNA-damaging agents. Δ*fghgg10* and Δ*fghgg13* exhibited altered sensitivity to all DNA damaged agents compared with the wild-type strain (Fig. 3F). Radial growth of Δ*fghgg10* and Δ*fghgg13* was markedly reduced on CM supplemented with camptothecin compared with the wild-type strain, and Δ*fghgg13* showed more severe growth defects compared with Δ*fghgg10*. The complemented strains of Δ*fghgg10* and Δ*fghgg13* restored growth in corresponding deletion mutants under DNA-damaging conditions. These results indicate that *FgHGG10* and *FgHGG13* play important roles in DNA repair when exposed to oxidative stress.

Furthermore, we investigated the role of *FgHGG4*, *FgHGG10*, and *FgHGG13* in asexual reproduction in *F. graminearum*. Compared with the wild type, the Δ*fghgg10* and Δ*fghgg13* mutants showed no altered phenotypes in conidiation. However, the deletion of *FgHGG4* resulted in increased conidial formation (Fig. S1A). Also, the conidia of Δ*fghgg4* were longer than that of the wild type (Fig. S1B and C). To determine the sexual reproduction, we cultured the wild type and those mutants on carrot agar media. The Δ*fghgg4* mutant produced perithecia normally. In contrast, no perithecia were formed by Δ*fghgg10* and Δ*fghgg13* mutants (Fig. S1D). These results support that the mechanisms that function importantly in responding to stress are also required for normal reproduction in fungi.

To evaluate the virulence of the three hub gene deletion mutants, conidial suspension of each strain was injected into the middle of the flowering wheat head. Whereas the wild-type strain caused normal blight symptoms on the wheat head, Δ*fghgg4*, Δ*fghgg10*, and Δ*fghgg13* showed markedly reduced virulence (Fig. 3G). To colonize a plant’s cells, fungi must endure host-derived oxidative bursts. Deletion of each gene caused a vulnerability to oxidative stress, which appeared to lead to a failure to overcome plant defenses. These results imply that the mechanisms associated with the oxidative stress response are also directly related to pathogenicity.

Moreover, we constructed a series of double-deletion mutants of *FgHGG4*, *FgHGG10*, and *FgHGG13* to identify whether they function simultaneously. The mutant construction was confirmed by PCR (Fig. S2A), and the sensitivity to oxidative stress was examined. Compared with the Δ*fghgg4* mutants, Δ*fghgg4*Δ*fghgg10* and Δ*fghgg4*Δ*fghgg13* strains showed increased sensitivity to oxidative stress (Fig. S2B and C). These results suggest that FgHgg4-mediated mechanisms function in oxidative stress resistance independently of the DNA repair system. Additionally, the growth inhibition rate of the Δ*fghgg10*Δ*fghgg13* is not significantly different from that of the Δ*fghgg13* mutant, indicating that FgHgg13 has a role in oxidative stress response overlapping FgHgg10 functions.

### Sensitivity of essential gene mutants to oxidative stress

**Fig 4:**
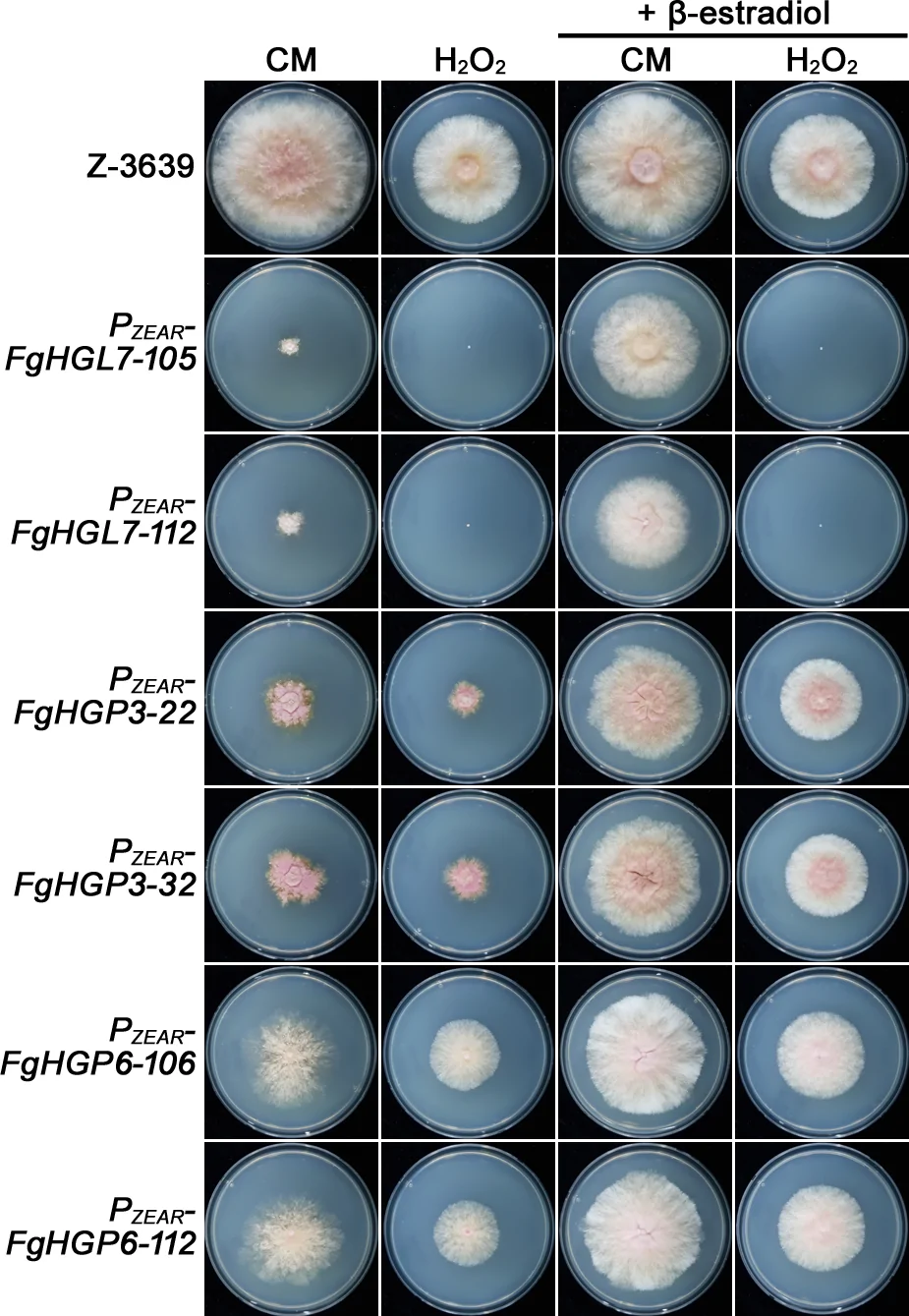
Oxidative stress sensitivity of promoter replacement mutants. Fungal strains were cultured on CM and CM supplemented with 10 mM H_2_O_2_, and mycelial growth was observed in the absence and presence of β-estradiol. Pictures were taken 5 days after inoculation.

We failed to delete *FgHGG12*, *FgHGL7*, *FgHGP3*, and *FgHGP6* using homologous recombination among 31 hub genes. These genes are considered essential to fungal viability, and essential genes can be novel targets for disease control. We therefore introduced conditional gene expression to those genes and confirmed the phenotype. In *F. graminearum*, zearalenone-inducible promoter (*P*_ZEAR_) was used for conditional gene expression and β-estradiol can substitute for zearalenone (61, 62). The promoter of each gene was replaced with *P*_ZEAR_ and the replacement was confirmed by Southern blot hybridization (Fig. S3). The promoters of *FgHGL7*, *FgHGP3*, and *FgHGP6* were successfully replaced with *P*_ZEAR_, and we observed vegetative growth of the generated mutants, both with and without supplementation of β-estradiol. Each mutant, *P*_ZEAR_ *-FgHGL7* in particular, exhibited severe growth defects on CM, while vegetative growth of the mutants recovered when they were grown on CM supplemented with β-estradiol (Fig. 4). To confirm the sensitivity of each strain to oxidative stress, all mutants were cultured in CM and treated with and without 10 mM H_2_O_2_. Repression or induction of *FgHGP3* and *FgHGP6* did not affect sensitivity to oxidative stress. In contrast, repression of *FgHGL7* resulted in hypersensitivity to oxidative stress, and partial induction of *FgHGL7* gene expression through β-estradiol treatment did not restore growth defects of *P*_ZEAR_ *-FgHGL7*. These results suggest that *FgHGL7* is essential for fungal viability, and that full expression of *FgHGL7* is required for growth under oxidative stress.

### Heme biosynthesis pathway is important for resistance to oxidative stress

**Fig 5:**
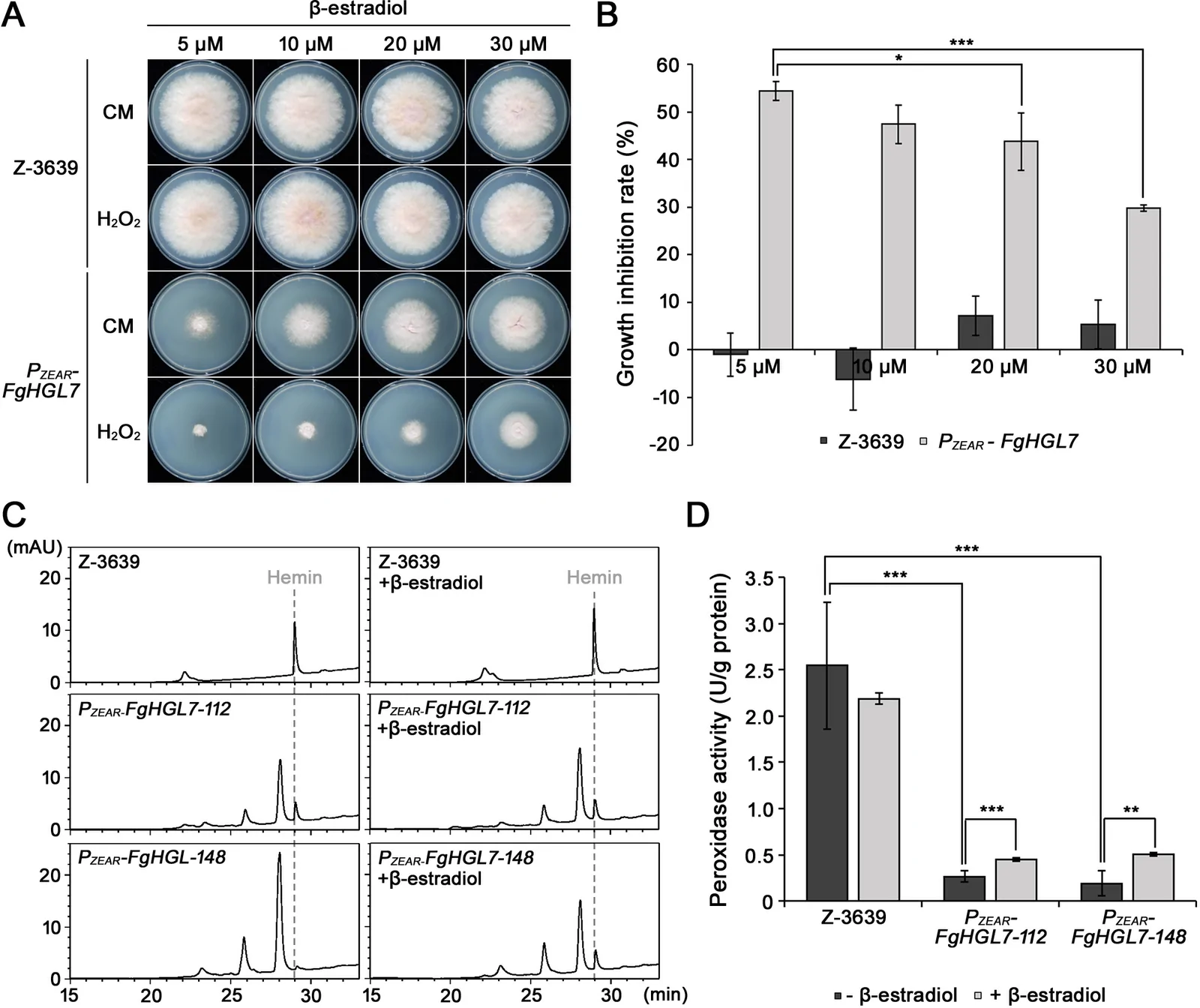
Heme biosynthesis is required for oxidative stress resistance. (**A**) Mycelial growth of the wild-type strain and *P_ZEAR_-FgHGL7* under oxidative stress conditions. Each strain was inoculated on CM and CM supplemented with 2 mM H_2_O_2_, and mycelial growth was observed at different concentrations of β-estradiol. Photographs were taken 5 days after inoculation. (**B**) Statistical analysis of mycelial growth inhibition under oxidative stress. (**C**) High-performance liquid chromatography profiling of hemin production by the wild-type strain and conditional *FgHGL7* expression mutants with and without addition of β-estradiol. The gray line represents hemin. (**D**) Peroxidase activity of the fungal strains. Asterisks represent significant differences from the wild type (*P < 0.05; **P < 0.01; ***P < 0.001; *t*-test).

To confirm whether repression of *FgHGL7* genes caused hypersensitivity to oxidative stress, we inoculated *P_ZEAR_-FgHGL7* mutants on CM supplemented with 2 mM H_2_O_2_ and observed mycelial growth at various concentrations of β-estradiol. As the concentration of β-estradiol increased, the inhibition rate of *P_ZEAR_-FgHGL7* decreased under oxidative stress (Fig. 5A and B), suggesting that expression of *FgHGL7* is positively correlated with resistance to oxidative stress in *F. graminearum*.

*FgHGL7* is predicted to encode coproporphyrinogen III oxidase, an enzyme that has a role in the biosynthetic pathway of heme, converting coproporphyrinogen III into protoporphyrinogen IX through oxidative decarboxylation (63, 64). It was reported that coproporphyrinogen III oxidase is involved in the regulation of intracellular heme levels in *Aspergillus niger* (65). To identify the effect of suppressing *FgHGL7* on heme biosynthesis in *F. graminearum*, the production of hemin/heme was determined in both wild-type and *P_ZEAR_-FgHGL7* mutant strains (Fig. 5C). In the wild-type strain, hemin production peaked at a retention time of 28.957 min, whereas significant reduction of peak areas was observed in *P_ZEAR_-FgHGL7* mutants. Rather, a high-performance liquid chromatography (HPLC) profile of *P_ZEAR_-FgHGL7* mutants showed two prominent peaks at retention times of 25.935 and 28.120 min, indicating the presence of heme intermediates. When β-estradiol was treated, the profiles of *P_ZEAR_-FgHGL7* mutants showed an increase in the area of the peak corresponding to hemin, indicating partial restoration of heme biosynthesis.

Several studies have reported that heme-containing peroxidases or catalases have an important role in the oxidative stress response (66 – 68). Especially, it was confirmed that the heme-containing peroxidase (Fca6, Fca7, and Fpx1) has an important role in oxidative stress response, affecting total peroxidase activity in *F. graminearum* (20). Based on previous studies, we aimed to investigate whether the defect in heme biosynthesis reduces the activity of peroxidases. We determined the activities of peroxidase in the wild-type and *P_ZEAR_-FgHGL7* mutant strains, and it revealed that the peroxidase activity of the *P_ZEAR_-FgHGL7* mutants was remarkably decreased compared with that of the wild-type strain (Fig. 5D). Treatment of β-estradiol slightly increased peroxidase activity in *P_ZEAR_-FgHGL7*, but the levels were still lower than those of the wild type, suggesting that the partial restoration of gene expression of *FgHGL7* through β-estradiol treatment was insufficient to fully restore its function. These findings indicate that *FgHGL7* plays a role in heme biosynthesis, and that disruption of heme biosynthesis due to *FgHGL7* suppression affects the heme-containing peroxidase activity.

### Oxidative stress resistance mechanism in *F. graminearum*

To successfully infect a host plant, fungal pathogens must tolerate and combat the oxidative stress induced by the host’s immune system (69). Various mechanisms are activated to protect the cell from host-derived ROS. In this study, we attempted to specify those mechanisms by analyzing the gene expression profiles of oxidative stress-sensitive strains in *F. graminearum*. WGCNA methods identified the gene groups most closely correlated with oxidative stress, and functional enrichment analysis of the key modules revealed that several mechanisms are upregulated against oxidative stress: DNA damage response, autophagy, and ubiquitin-proteasome system.

Oxidative damage in DNA is repaired by the DNA damage response system, which prevents replication of defective DNA (38). DNA repair system and oxidative stress response appear to be closely associated. According to phenotypes described in FgTFPD (43), 4 of the 16 transcription factor mutants associated with DNA damage response in *F. graminearum* are sensitive to oxidative stress (70). Additionally, phenotypic analysis of the hub gene mutants revealed that the *FgHGG10-* and *FgHGG13*-encoding yeast orthologs Rad54 and Rad52, respectively, play a role in oxidative stress response, and these findings indicate that DNA repair system is essential for tolerance under oxidative stress.

Autophagy and ubiquitin-mediated proteasome systems contribute to the elimination of damaged cellular components (71). Autophagy has a role in the removal of damaged cytosolic components, and ubiquitin-mediated proteasomes selectively decompose damaged proteins (42). The ubiquitin-proteasome system and the 26S proteasome, in particular, are easily damaged by oxidative stress, but H_2_O_2_ triggers the disassembly of 26S proteasomes into 19S particles and 20S catalytic cores, and a 20S core can actively recognize and degrade oxidized protein (72). Autophagy and the UPS have been investigated in plant pathogenic fungi, and several studies have revealed that autophagy and UPS-related components, including peroxin and ubiquitin-like protein, are required for oxidative stress resistance (73 – 77). Collectively, as protein oxidations caused by oxidative stress lead to cellular dysfunction (78, 79), those mechanisms are essential to tolerating oxidative stress by controlling protein quality.

In this study, we identified core genes of each module and used them as tools to reveal the mechanisms related to oxidative stress response. Phenotypic analysis under the oxidative stress for 27 hub gene mutants identified *FgHGG4*, a subunit of the elongator complex, along with *FgHGG10* and *FgHGG13*. The elongator complex has a role in transcriptional elongation and is involved in wobble modification of tRNA (80, 81). In yeast, the chemical modification of wobble base uridines of tRNA reportedly affects the translational regulation of genes involved in oxidative stress responses (56). It has also been reported that Elp3, the other subunit of the elongator complex, has a role in the oxidative stress response in *F. graminearum* (58). However, the role of the elongator complex subunits in oxidative stress response varies among fungal species (82 – 84), and further studies are required on the specific regulatory mechanisms of the elongator complex in the oxidative stress response in *F. graminearum*.

Among the hub genes, four were identified as essential for fungal viability. We determined that *FgHGL7* is necessary for resistance to oxidative stress. Functional characterization of *FgHGL7* revealed that heme biosynthesis is required for an effective response to oxidative stress. Heme is an iron-containing tetrapyrrole ring with an essential role in diverse biological functions, serving as the prosthetic group of hemeproteins (85 – 87). In *F. graminearum*, 31 putative peroxidases were identified, 23 of which contain heme (20). Multiple studies have reported the role of peroxidases in ROS detoxification and oxidative stress responses (88 – 90). Cytochromes P450s (CYPs) are also representative heme-containing proteins, and act as monooxygenases that catalyze the oxidation of cellular substrates (91, 92). However, none of the CYP mutants reportedly display an altered phenotype under oxidative stress in *F. graminearum* (93). These indicate that heme is required for resistance to oxidative stress as a component of heme-containing peroxidase.

Collectively, a combined transcriptomic and physiological analysis identified the several mechanisms required for oxidative stress adaptation in *F. graminearum* including “DNA repair,” “protein degradation mechanisms,” “transcriptional elongation,” and “heme biosynthesis.” Furthermore, this study confirms the significance of oxidative stress resistance for pathogenicity and provides a comprehensive understanding of the mechanisms to cope with oxidative stress in plant pathogenic fungi.

## MATERIALS AND METHODS

### Fungal strains and growth conditions

The *F. graminearum* Z-3639 wild-type strain and the mutants used in this study were stored as mycelia in 20% glycerol at −80°C. All media were prepared as described in The *Fusarium* Laboratory Manual (94). Fungal strains were grown at 25°C.

### Nucleic acid manipulation and Southern blotting

Fungal genomic DNA was extracted with a cetyltrimethylammonium bromide protocol according to The *Fusarium* Laboratory Manual (94). Restriction endonuclease digestion, agarose gel electrophoresis, and gel blotting were performed following standard protocols (95). A North2South Biotin Random Prime Labeling Kit and a Chemiluminescent Hybridization and Detection Kit (Thermo Scientific, USA) were used for Southern blot hybridization. Total RNA was extracted using an Easy-Spin Total RNA Extraction Kit (Intron Biotech, Korea).

### RNA-seq and WGCNA analysis

Conidia suspensions were cultured in liquid CM at 25°C for 24 h with shaking. Fresh mycelia were shifted to liquid CM with or without 5 mM H_2_O_2_ and cultured for 30 min. Total RNA was extracted from harvested mycelia, and RNA-seq was performed using an Illumina HiSeq 2000 system.

Adapter sequences of reads derived from RNA-seq were trimmed in the Cutadapt program (96). Unreliable low-quality reads were removed from the raw reads using the FASTX-Toolkit if the average quality within 50% of the read sequences falls below a Phred score threshold of 28. Filtered clean reads were then mapped to reference genome sequences downloaded from the National Center for Biotechnology Information using the Burrows-Wheeler Aligner (BWA) program (97). Mapped reads per gene were counted by featureCounts tool v1.5.0 (98) and normalized using the reads per kilobase per million mapped reads (RPKM) method (99).

Highly co-expressed gene modules were inferred from total genes according to WGCNA tutorials (100, 101). A matrix of pairwise Pearson correlations between all genes was calculated and transformed into a pairwise adjacency matrix by a soft power threshold (β) derived from the pickSoftThreshold algorithm. The β of 12 powers satisfied the minimum value required to generate a scale-free topology network (a *R* ^2^ value ≥0.8). The pairwise adjacency matrix was re-transformed into a topological overlap measure to capture a co-expression relationship between genes in the network. Modules as groups of co-expressed genes were defined using the cutreeDynamic algorithm (deepSplit = 4) with a minimum module size of 200. MEDissThres was set to 0.7 to merge similar modules. The closely correlated modules with traits were selected by calculating the relevance between traits and a module eigengene for further analysis. Potential hub genes as highly interconnected genes within the module were estimated with a connectivity weight threshold of 0.2 in the co-expression network.

KEGG and GO enrichment analyses were conducted to explore the biological significance of the genes in each module. KEGG pathway data were downloaded from the KEGG database (http://www.genome.jp/kegg) and GO terms were obtained from the UniProt database (https://www.uniprot.org). The statistical significance of enrichment was determined using a hypergeometric distribution (*P* < 0.05).

### Genetic manipulations and fungal transformations

For gene deletion, 5´- and 3´- flanking regions of the open reading frame (ORF) were amplified from the genomic DNA of an *F. graminearum* wild-type strain. A geneticin resistance gene cassette (*GEN*) was amplified from a plasmid pII99. Three amplicons were fused, and final PCR products were created by double-joint (DJ) PCR methods (102). To complement the Δ*fghgg10* and Δ*fghgg13* deletion mutants (*FgHGG10*com, *FgHGG13*com), the construct that included a native promoter region of the gene, the ORF, and the 3´- flanking region of the ORF was amplified. A PCR product including the green fluorescence protein and a hygromycin resistance cassette (*GFP-HYG*) was amplified from the plasmid pIGPAPA. The final PCR products were transformed into the deletion strains.

For *FgHGG4* and other putative essential gene deletions, a CRISPR-Cas9–mediated transformation was conducted to increase deletion efficiency (52). Briefly, 5´- flanking of the ORF was amplified with primers containing the optimal cleavage site. 3´- Flankings of the ORF and *GEN* cassette were amplified, and the final PCR products were constructed as described earlier. Before fungal transformation, synthesized sgRNA (Macrogen, Korea) and purchased Cas9 protein (Thermo Scientific, USA) were preassembled and added into protoplasts with the resulting amplicons.

For complementation of the Δ*fghgg4*, the ORF of *FgHGG4* and its putative promoter region (approximately 1 kb upstream of the transcription start site) were amplified. The PCR fragments were co-transformed with a pDL2/*XhoI* (103) linearized plasmid into yeast strain PJ69-4A. The resulting plasmid was extracted using Zymoprep Yeast Plasmid Miniprep II (Zymo Research, USA) and transformed into an *Escherichia coli* DH10B strain. After verification of the sequence, a recombinant plasmid was extracted and linearized for fungal transformation.

To generate a conditional gene expression mutant (*P_ZEAR_* mutants), the promoter regions of the target genes were replaced with *P_ZEAR_*. The hygromycin resistance gene (*HYG*)-*P_ZEAR_* cassette was amplified from the *P_ZEAR_-FgHSP90* strains (104). The 5´- and 3´- flanking regions of the target genes were amplified and fused with *HYG-P_ZEAR_* cassette using DJ PCR methods (102). After amplifying with the nested primers, the final amplicons were used for transformation.

Each final PCR product or linearized plasmid was transformed into protoplasts using polyethylene glycol-mediated transformation, and all mutations were confirmed with Southern blot hybridization analysis (Fig. S3). Primers used in this study were synthesized by an oligonucleotide synthesis facility (Bioneer, Korea) and listed in Table S2.

### Quantitative real-time PCR

First-strand cDNA was synthesized from total RNA using SuperScript III reverse transcriptase (Invitrogen, USA) following the manufacturer’s instructions. Quantitative real-time PCR amplification was conducted with iTaq Universal SYBR Green Supermix (Bio-Rad, USA) and a CFX real-time PCR system (Bio-Rad). PCR amplification was performed three times with three biological replicates, and the relative transcript levels of the target gene were calculated as described previously (105). The primers used for qRT-PCR are listed in Table S2.

### Virulence tests

Virulence tests were conducted using the wheat cultivar Eunpamil as previously described (106). Each fungal strain was cultured in 25 mL of carboxymethylcellulose (CMC) at 25°C for 5 days. Harvested conidia were resuspended in 0.01% Tween 20 solution. A 10 µL suspension of conidia (10^6^ conidia/mL) was injected into the middle of the spikelet. The inoculated wheat heads were enveloped with a plastic bag to create humid conditions for 3 days. After an additional 11 days, the symptoms were observed and the number of infected spikelets was measured. Virulence tests were conducted with 10 replicate inoculations for each fungal strain.

### Asexual and sexual reproduction assays

For the conidiation assay, 10 mycelial plugs of each strain were inoculated in 30 mL CMC media for 5 days. Conidia were counted with a hemocytometer. The experiment was repeated three times.

For the examination of sexual reproduction, each strain was grown on carrot agar media for 5 days, and aerial hyphae were removed with 400 µL of a 2.5% Tween 60 solution to induce sexual reproduction. The resulting media were incubated under near-UV light (Sankyo Denki, Tokyo, Japan), and the perithecia formation was observed 7 days after induction.

### Heme quantification using HPLC

Heme quantification was conducted as described previously (65, 107, 108). Briefly, frozen mycelia were ground into fine powder with liquid nitrogen. Samples were homogenized in phosphate-buffered saline, and protein contents were determined by the Bradford method (109). A portion of extract was mixed with an equal volume of acetone and concentrated HCl (97.5/2.5; vol/vol). After centrifuging, the supernatant was analyzed by reversed-phase HPLC with an ultraviolet detector.

The analysis was carried out using a Shimadzu UFLC HPLC system (Shimadzu, Japan). To prepare solvent A, 0.1 M ammonium phosphate solution (pH adjusted to 3.5 with phosphoric acid) was filtered with a 0.45-µm membrane filter (Millipore, USA). The filtered solution was mixed with methanol (56:44; vol/vol), and the pH of the solution was adjusted to 3.4 with phosphoric acid. Pure methanol was used for solvent B. Samples were eluted on a C18 HPLC column (Shimadzu, Japan) at 35°C at a flow rate of 1.5 mL/min. In gradient elution, the composition of solvent B was changed from 30% to 100% for 15 min. The spectra of the samples were monitored at 405 nm.

### Estimation of peroxidase enzyme activity

Total peroxidase activity assay was performed using a QuantiChrom peroxidase assay (BioAssay Systems, USA) as previously described (20). Crude proteins were extracted, and the concentration was estimated with a Bradford assay. Further steps followed the manufacturer’s instructions.

## Data Availability

The RNA-seq data have been deposited in the NCBI database under the BioProject accession number PRJNA937405.

## SUPPLEMENTAL MATERIAL

Fig. S1 to S3 spectrum.01485-23-s0001.pdf Conidiation and sexual reproduction of Δ*fghgg4*, Δ*fghgg10*, and Δ*fghgg13*. Sensitivity of double deletion mutants to oxidative stress. Confirmation of the genetic manipulation by southern blot analyses.

Table S1 spectrum.01485-23-s0002.docx GO enrichment analysis for the module genes.

Table S2 spectrum.01485-23-s0003.docx Primers used in this study.

ASM does not own the copyrights to Supplemental Material that may be linked to, or accessed through, an article. The authors have granted ASM a non-exclusive, world-wide license to publish the Supplemental Material files. Please contact the corresponding author directly for reuse.
