# Supplementary material for: A combined transcriptomic and physiological approach to understanding the adaptive mechanisms to cope with oxidative stress in Fusarium graminearum
Source: Microbiol Spectr. 2023 Sep 6;11(5):e01485-23. doi: 10.1128/spectrum.01485-23 (PMC10581207; doi:10.1128/spectrum.01485-23)
Supplement: Fig. S1 to S3 — Conidiation and sexual reproduction of Δfghgg4, Δfghgg10, and Δfghgg13. Sensitivity of double deletion mutants to oxidative stress. Confirmation of the genetic manipulation by southern blot analyses. [file spectrum.01485-23-s0001.pdf]

Supplemental material

A combined transcriptomic and physiological approach to understanding the adaptive mechanisms to cope with oxidative stress in *Fusarium graminearum*

Jiyeun Park[1#], Hyun-Hee Lee[2#], Heeji Moon[1], Nahyun Lee[1], Sieun Kim[1], Jung-Eun Kim[3], Yoonji Lee[1], Kyunghun Min[1], Hun Kim[4], Gyung Ja Choi[4], Yin-Won Lee[1], Young-Su Seo[2]* and Hokyoung Son[1,5]*

[1]Department of Agricultural Biotechnology, Seoul National University, Seoul, 08826, Republic of Korea

[2]Department of Integrated Biological Science, Pusan National University, Busan, 46247, Republic of Korea

[3]Research Institute of Climate Change and Agriculture, National Institute of Horticultural and Herbal Science, Jeju, Republic of Korea

[4]Center for Eco-friendly New Materials, Korea Research Institute of Chemical Technology, Daejeon, 34114, Republic of Korea

[5]Research Institute of Agriculture and Life Sciences, Seoul National University, Seoul, 08826, Republic of Korea

[#]Jiyeun Park and Hyun-Hee Lee contributed equally to this work.

Address correspondence to Hokyoung Son, hogongi7@snu.ac.kr, or Young-Su Seo, yseo2011@pusan.ac.kr.

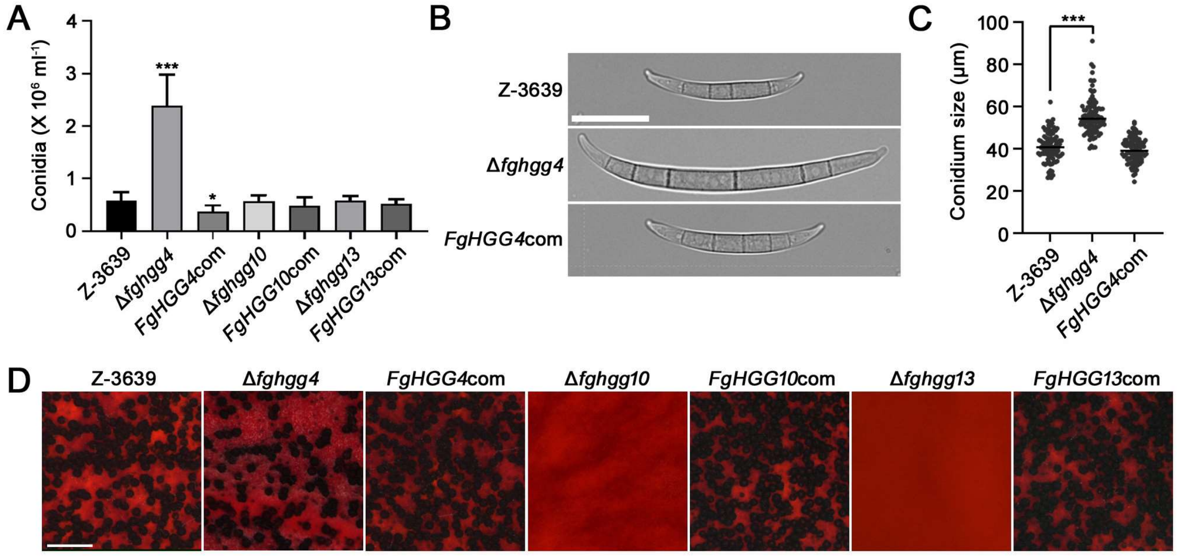

Fig. S1. Conidiation and sexual reproduction of Δ*fghgg4*, Δ*fghgg10*, and Δ*fghgg13*. (A) Conidiation of the Δ*fghgg4*, Δ*fghgg10* and Δ*fghgg13*. Each strain was cultured on carboxymethyl cellulose (CMC) during five days, and the number of conidia was counted. (B) Conidia morphology of wild-type, Δ*fghgg4*, and complemented strains. Scale bar = 20 μm. (C) Statistical analysis of conidial length. A total of 100 conidia was observed per examination. Asterisks represent significant differences from the wild type (***$P < 0.001$; *t*-test). (D) Perithecium formation. Photographs were taken 7 days after sexual induction. Scale bar = 1000 μm.

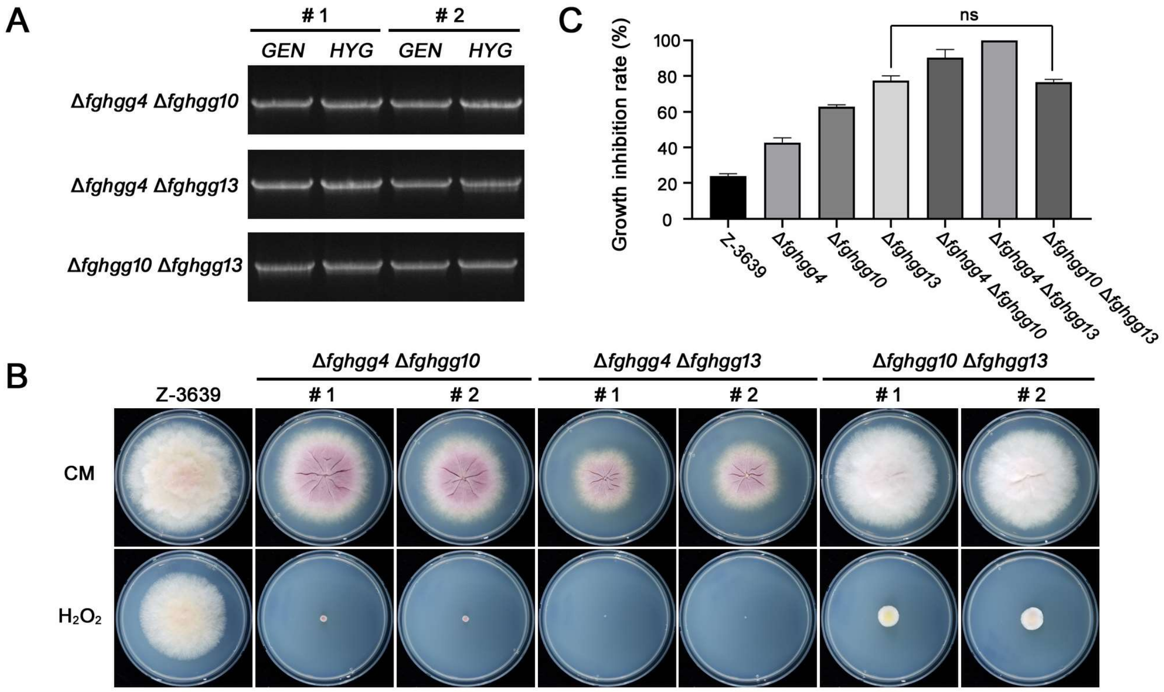

Fig. S2. Sensitivity of double deletion mutants to oxidative stress. (A) PCR-based confirmation of the double deletion mutant strains of *F. graminearum*. For the construction of double deletion mutants, the open reading frame of target genes was replaced with the hygromycin resistance cassette in the single deletion mutant. PCR analysis was conducted on two isolates. Lane 1 and 3 indicate the amplification of the geneticin resistance cassette (*GEN*), and Lane 2 and 4 indicate the amplification of the hygromycin resistance cassette (*HYG*) from the constructed mutant. (B) Mycelial growth of six oxidative stress-sensitive mutants under oxidative stress. Each strain was inoculated on complete medium (CM) and CM supplemented with 10 mM H_2O_2. Photographs were taken 5 days after inoculation. (C) Statistical analysis of mycelia growth inhibition under oxidative stress. ns, not significant (P>0.05; t-test).

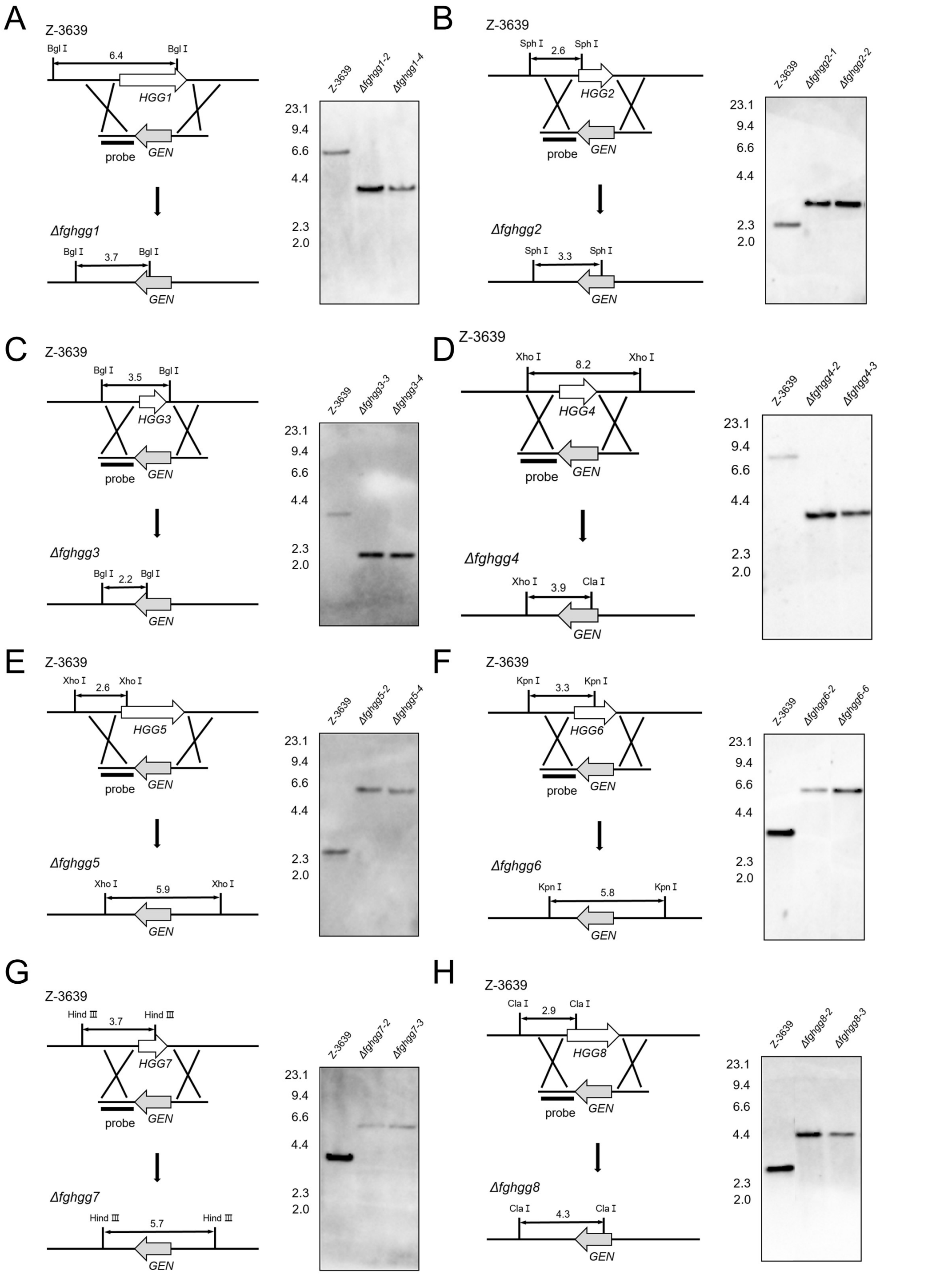

Fig. S3. Confirmation of the genetic manipulation by southern blot analyses. (A-AF) The left and right sides of each panel show the strategy used for genetic manipulation, and Southern blot analysis, respectively. The size of the DNA standards (kb) and restriction enzymes used for each blot are indicated on the strategy.

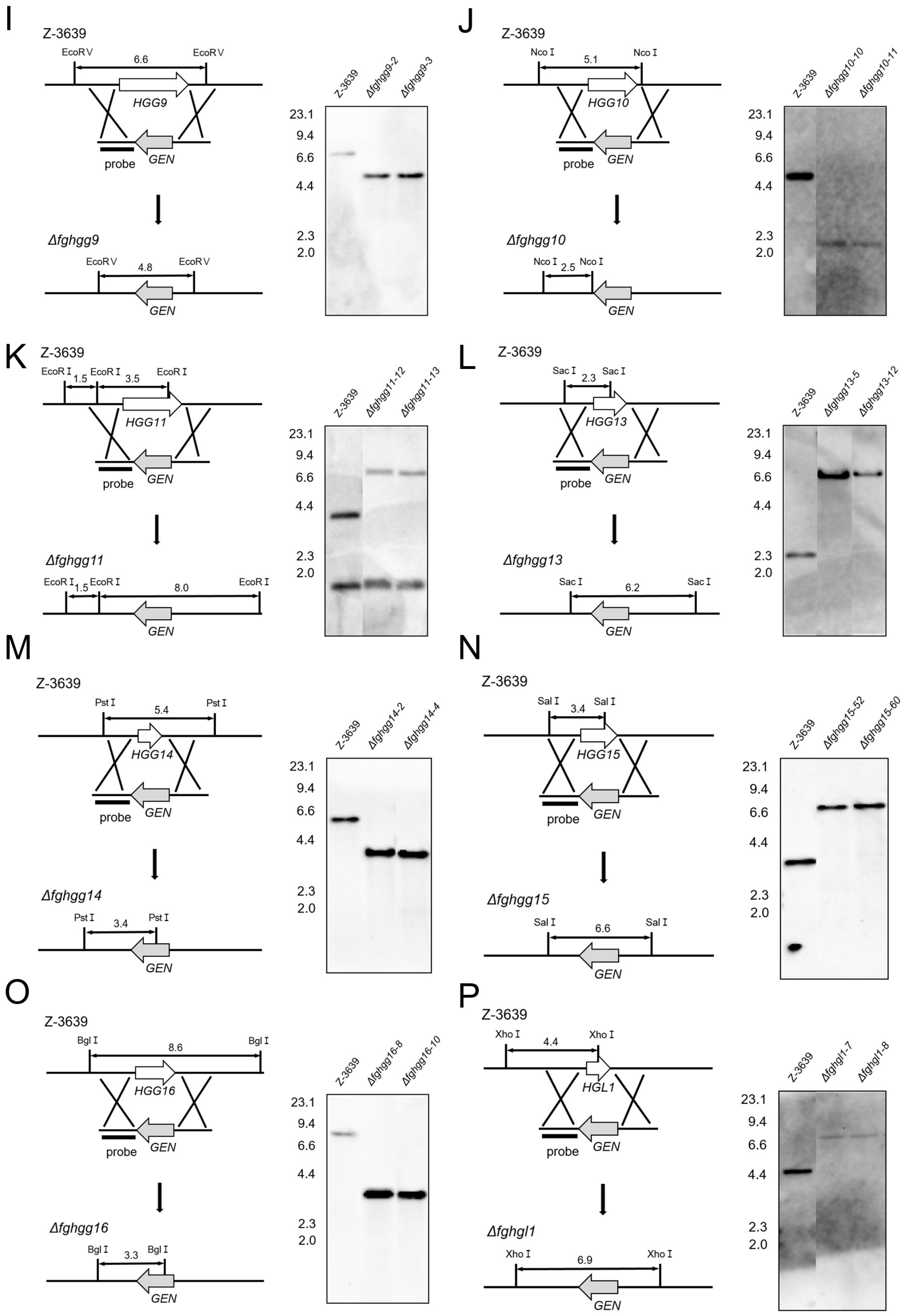

I
Z-3639
EcoR V
6.6
EcoR V
HGG9
probe
GEN
Δfghgg9
EcoR V
4.8
EcoR V
GEN
Z-3639
Δfghgg9-2
Δfghgg9-3
23.1
9.4
6.6
4.4
2.3
2.0
J
Z-3639
Nco I
5.1
Nco I
HGG10
probe
GEN
Δfghgg10
Nco I
2.5
Nco I
GEN
Z-3639
Δfghgg10-10
Δfghgg10-11
23.1
9.4
6.6
4.4
2.3
2.0
K
Z-3639
EcoR I
1.5
EcoR I
3.5
EcoR I
HGG11
probe
GEN
Δfghgg11
EcoR I
1.5
EcoR I
8.0
EcoR I
GEN
Z-3639
Δfghgg11-12
Δfghgg11-13
23.1
9.4
6.6
4.4
2.3
2.0
L
Z-3639
Sac I
2.3
Sac I
HGG13
probe
GEN
Δfghgg13
Sac I
6.2
Sac I
GEN
Z-3639
Δfghgg13-5
Δfghgg13-12
23.1
9.4
6.6
4.4
2.3
2.0
M
Z-3639
Pst I
5.4
Pst I
HGG14
probe
GEN
Δfghgg14
Pst I
3.4
Pst I
GEN
Z-3639
Δfghgg14-2
Δfghgg14-4
23.1
9.4
6.6
4.4
2.3
2.0
N
Z-3639
Sal I
3.4
Sal I
HGG15
probe
GEN
Δfghgg15
Sal I
6.6
Sal I
GEN
Z-3639
Δfghgg15-52
Δfghgg15-60
23.1
9.4
6.6
4.4
2.3
2.0
O
Z-3639
Bgl I
8.6
Bgl I
HGG16
probe
GEN
Δfghgg16
Bgl I
3.3
Bgl I
GEN
Z-3639
Δfghgg16-8
Δfghgg16-10
23.1
9.4
6.6
4.4
2.3
2.0
P
Z-3639
Xho I
4.4
Xho I
HGL1
probe
GEN
Δfghgl1
Xho I
6.9
Xho I
GEN
Z-3639
Δfghgl1-7
Δfghgl1-8
23.1
9.4
6.6
4.4
2.3
2.0

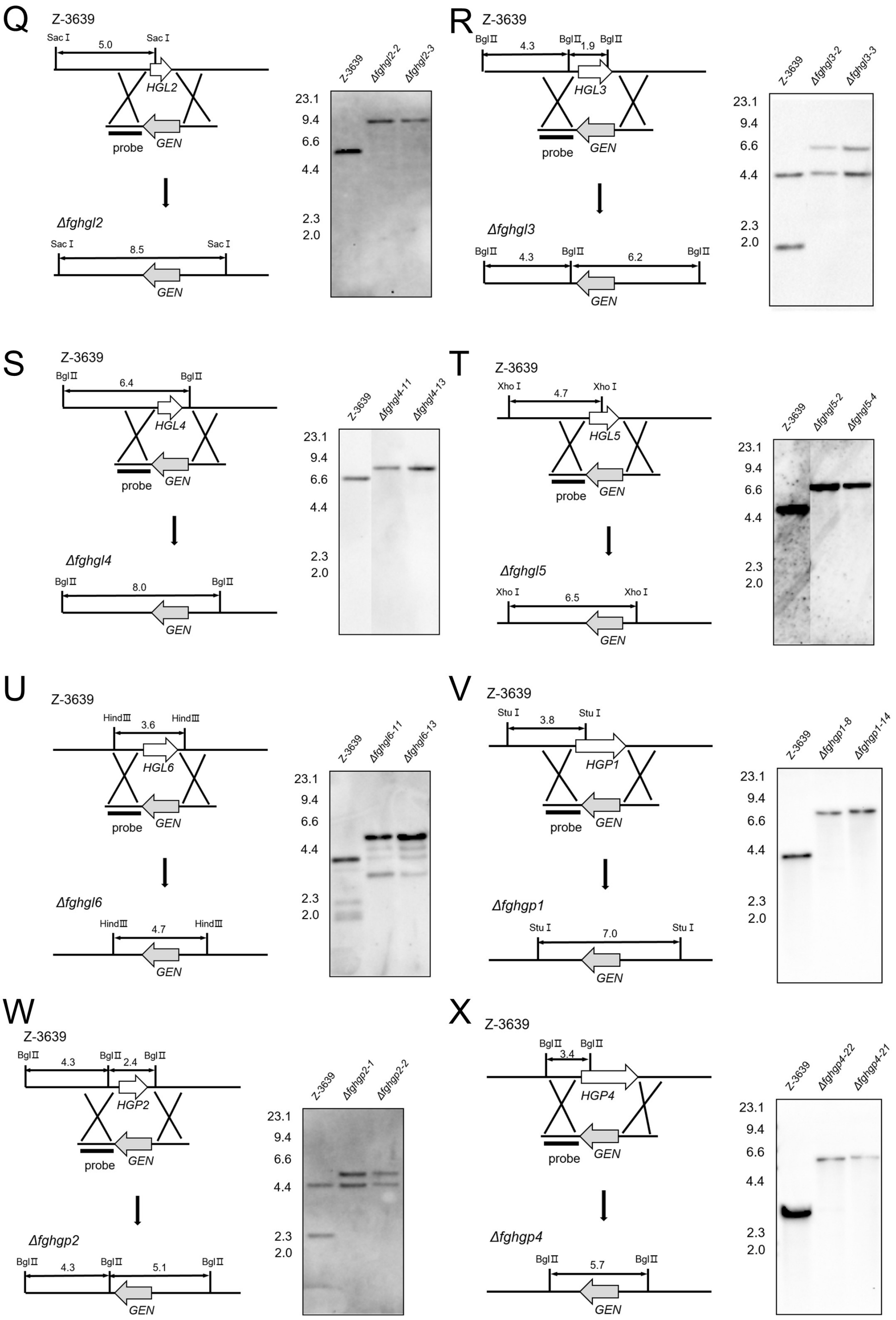
Q
Z-3639
Sac I
5.0
Sac I
HGL2
probe
GEN
Δfghgl2
Sac I
8.5
Sac I
GEN
Z-3639
Δfghgl2-2
Δfghgl2-3
23.1
9.4
6.6
4.4
2.3
2.0
R
Z-3639
BglII
4.3
BglII
1.9
BglII
HGL3
probe
GEN
Δfghgl3
BglII
4.3
BglII
6.2
BglII
GEN
Z-3639
Δfghgl3-2
Δfghgl3-3
23.1
9.4
6.6
4.4
2.3
2.0
S
Z-3639
BglII
6.4
BglII
HGL4
probe
GEN
Δfghgl4
BglII
8.0
BglII
GEN
Z-3639
Δfghgl4-11
Δfghgl4-13
23.1
9.4
6.6
4.4
2.3
2.0
T
Z-3639
Xho I
4.7
Xho I
HGL5
probe
GEN
Δfghgl5
Xho I
6.5
Xho I
GEN
Z-3639
Δfghgl5-2
Δfghgl5-4
23.1
9.4
6.6
4.4
2.3
2.0
U
Z-3639
HindIII
3.6
HindIII
HGL6
probe
GEN
Δfghgl6
HindIII
4.7
HindIII
GEN
Z-3639
Δfghgl6-11
Δfghgl6-13
23.1
9.4
6.6
4.4
2.3
2.0
V
Z-3639
Stu I
3.8
Stu I
HGP1
probe
GEN
Δfghgp1
Stu I
7.0
Stu I
GEN
Z-3639
Δfghgp1-8
Δfghgp1-14
23.1
9.4
6.6
4.4
2.3
2.0
W
Z-3639
BglII
4.3
BglII
2.4
BglII
HGP2
probe
GEN
Δfghgp2
BglII
4.3
BglII
5.1
BglII
GEN
Z-3639
Δfghgp2-1
Δfghgp2-2
23.1
9.4
6.6
4.4
2.3
2.0
X
Z-3639
BglII
3.4
BglII
HGP4
probe
GEN
Δfghgp4
BglII
5.7
BglII
GEN
Z-3639
Δfghgp4-22
Δfghgp4-21
23.1
9.4
6.6
4.4
2.3
2.0

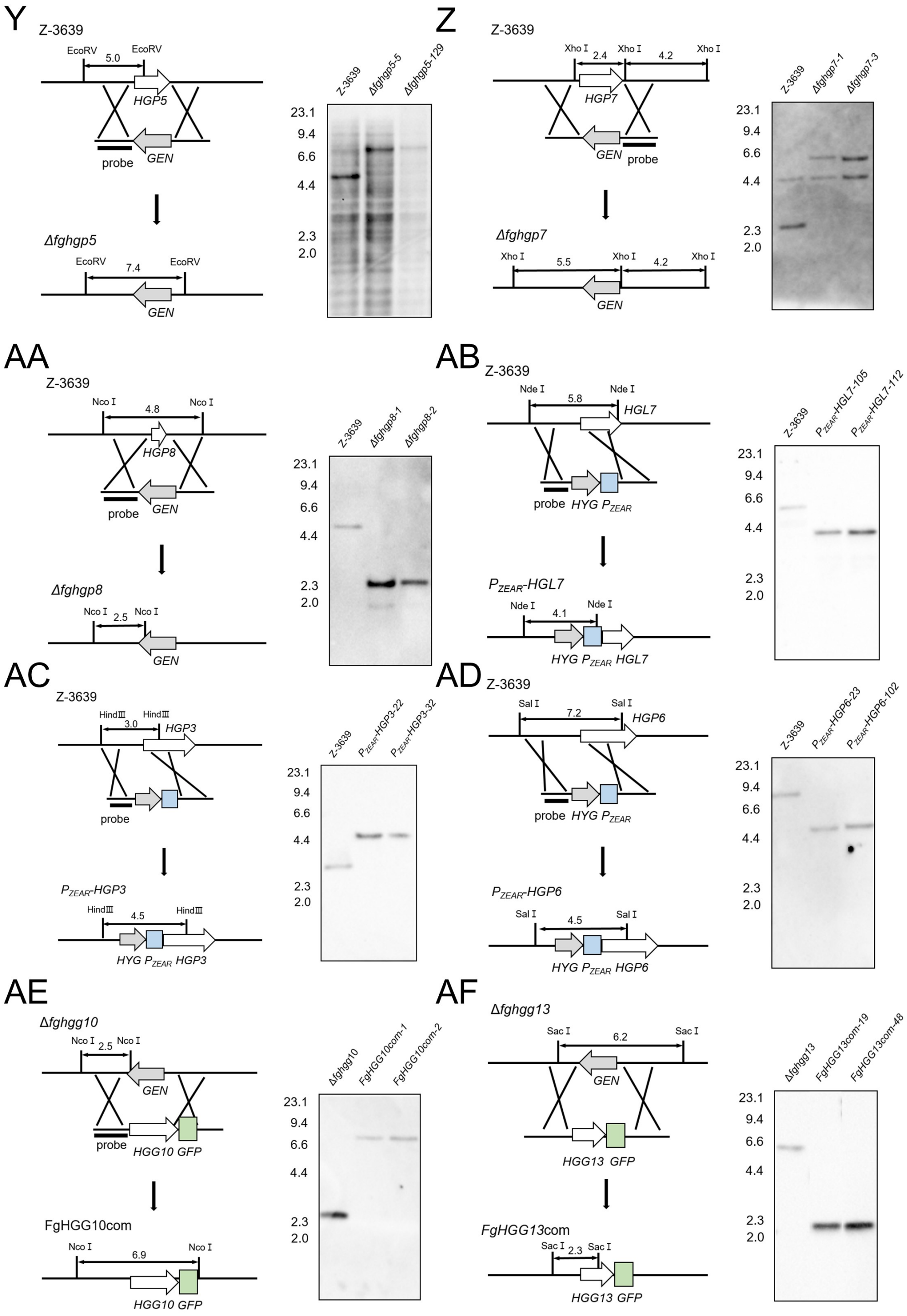

Y
Z-3639
EcoRV
5.0
EcoRV
HGP5
probe
GEN
Δfghgp5
EcoRV
7.4
EcoRV
GEN
Z-3639
Δfghgp5-5
Δfghgp5-129
23.1
9.4
6.6
4.4
2.3
2.0
Z
Z-3639
Xho I
2.4
Xho I
4.2
Xho I
HGP7
GEN
probe
Δfghgp7
Xho I
5.5
Xho I
4.2
Xho I
GEN
Z-3639
Δfghgp7-1
Δfghgp7-3
23.1
9.4
6.6
4.4
2.3
2.0
AA
Z-3639
Nco I
4.8
Nco I
HGP8
probe
GEN
Δfghgp8
Nco I
2.5
Nco I
GEN
Z-3639
Δfghgp8-1
Δfghgp8-2
23.1
9.4
6.6
4.4
2.3
2.0
AB
Z-3639
Nde I
5.8
Nde I
HGL7
probe
HYG P_ZEAR
P_ZEAR-HGL7
Nde I
4.1
Nde I
HYG P_ZEAR HGL7
Z-3639
P_ZEAR-HGL7-105
P_ZEAR-HGL7-112
23.1
9.4
6.6
4.4
2.3
2.0
AC
Z-3639
HindIII
3.0
HindIII
HGP3
probe
P_ZEAR-HGP3
HindIII
4.5
HindIII
HYG P_ZEAR HGP3
Z-3639
P_ZEAR-HGP3-22
P_ZEAR-HGP3-32
23.1
9.4
6.6
4.4
2.3
2.0
AD
Z-3639
Sal I
7.2
Sal I
HGP6
probe
HYG P_ZEAR
P_ZEAR-HGP6
Sal I
4.5
Sal I
HYG P_ZEAR HGP6
Z-3639
P_ZEAR-HGP6-23
P_ZEAR-HGP6-102
23.1
9.4
6.6
4.4
2.3
2.0
AE
Δfghgg10
Nco I
2.5
Nco I
GEN
probe
HGG10 GFP
FgHGG10com
Nco I
6.9
Nco I
HGG10 GFP
Δfghgg10
FgHGG10com-1
FgHGG10com-2
23.1
9.4
6.6
4.4
2.3
2.0
AF
Δfghgg13
Sac I
6.2
Sac I
GEN
HGG13 GFP
FgHGG13com
Sac I
2.3
Sac I
HGG13 GFP
Δfghgg13
FgHGG13com-19
FgHGG13com-48
23.1
9.4
6.6
4.4
2.3
2.0
