# Supplementary material for: A combined transcriptomic and physiological approach to understanding the adaptive mechanisms to cope with oxidative stress in Fusarium graminearum
Source: Microbiol Spectr. 2023 Sep 6;11(5):e01485-23. doi: 10.1128/spectrum.01485-23 (PMC10581207; doi:10.1128/spectrum.01485-23)
Supplement: Table S1 — GO enrichment analysis for the module genes. [file spectrum.01485-23-s0002.docx]

**Table. S1.** GO enrichment analysis for the module genes

| Module | GO ID | Term | Count | P-value |
| --- | --- | --- | --- | --- |
| Greenyellow | GO:0006281 | DNA repair | 27 | 2.39E-13 |
|  | GO:0006310 | DNA recombination | 8 | 9.79E-07 |
|  | GO:0000723 | telomere maintenance | 4 | 4.11E-04 |
|  | GO:0009073 | aromatic amino acid family biosynthetic process | 4 | 1.14E-03 |
|  | GO:0000105 | histidine biosynthetic process | 4 | 2.45E-03 |
|  | GO:1902600 | proton transmembrane transport | 6 | 5.55E-03 |
|  | GO:0006289 | nucleotide-excision repair | 5 | 5.71E-03 |
|  | GO:0006355 | regulation of transcription, DNA-templated | 51 | 7.10E-03 |
|  | GO:0000724 | double-strand break repair via homologous recombination | 2 | 9.47E-03 |
|  | GO:0045116 | protein neddylation | 2 | 9.47E-03 |
|  | GO:0006303 | double-strand break repair via nonhomologous end joining | 2 | 9.47E-03 |
|  | GO:0006974 | cellular response to DNA damage stimulus | 2 | 9.47E-03 |
|  | GO:0006420 | arginyl-tRNA aminoacylation | 2 | 9.47E-03 |
|  | GO:0006367 | transcription initiation from RNA polymerase II promoter | 4 | 1.16E-02 |
|  | GO:0006511 | ubiquitin-dependent protein catabolic process | 8 | 1.19E-02 |
|  | GO:0002098 | tRNA wobble uridine modification | 3 | 1.47E-02 |
|  | GO:0043161 | proteasome-mediated ubiquitin-dependent protein catabolic process | 2 | 2.66E-02 |
|  | GO:0006419 | alanyl-tRNA aminoacylation | 2 | 2.66E-02 |
|  | GO:0034227 | tRNA thio-modification | 2 | 2.66E-02 |
|  | GO:0006302 | double-strand break repair | 2 | 2.66E-02 |
|  | GO:0051103 | DNA ligation involved in DNA repair | 2 | 2.66E-02 |
|  | GO:0006260 | DNA replication | 7 | 4.85E-02 |
|  | GO:0006139 | nucleobase-containing compound metabolic process | 3 | 4.94E-02 |
|  | GO:0006891 | intra-Golgi vesicle-mediated transport | 2 | 4.97E-02 |
|  | GO:0006821 | chloride transport | 2 | 4.97E-02 |
| Lightcyan | GO:0008610 | lipid biosynthetic process | 3 | 1.53E-03 |
|  | GO:0055114 | oxidation-reduction process | 28 | 3.08E-03 |
|  | GO:0071423 | malate transmembrane transport | 2 | 6.86E-03 |
|  | GO:0006520 | cellular amino acid metabolic process | 3 | 1.35E-02 |
| Purple | GO:0006468 | protein phosphorylation | 16 | 1.28E-04 |
|  | GO:0015986 | ATP synthesis coupled proton transport | 5 | 1.72E-04 |
|  | GO:0000079 | regulation of cyclin-dependent protein serine/threonine kinase activity | 4 | 2.46E-04 |
|  | GO:0006633 | fatty acid biosynthetic process | 3 | 1.64E-03 |
|  | GO:0006044 | N-acetylglucosamine metabolic process | 2 | 5.90E-03 |
|  | GO:0006096 | glycolytic process | 3 | 6.23E-03 |
|  | GO:0046034 | ATP metabolic process | 2 | 1.15E-02 |
|  | GO:0008654 | phospholipid biosynthetic process | 3 | 1.48E-02 |
|  | GO:0009082 | branched-chain amino acid biosynthetic process | 2 | 1.85E-02 |
|  | GO:0007005 | mitochondrion organization | 2 | 2.70E-02 |
