# Supplementary material for: A combined transcriptomic and physiological approach to understanding the adaptive mechanisms to cope with oxidative stress in Fusarium graminearum
Source: Microbiol Spectr. 2023 Sep 6;11(5):e01485-23. doi: 10.1128/spectrum.01485-23 (PMC10581207; doi:10.1128/spectrum.01485-23)
Supplement: Table S2 — Primers used in this study. [file spectrum.01485-23-s0003.docx]

**Table. S2.** Primers used in this study

| Primer name | Sequence (5’→3’) | Description |
| --- | --- | --- |
| HGG1 5F | CGTCAAAGTCTTCCTCATCAGCAA | For *FgHGG1* deletion |
| HGG1 5N | CGTCCTTTGTGGTCTCGCTCTATT |  |
| HGG1 5R | gcacaggtacacttgtttagagCTTTCTCTTTGATGGCGGTGTTA |  |
| HGG1 3F | ccttcaatatcatcttctgtcgTACTTGTTGGTTGCGTACAGGATGT |  |
| HGG1 3N | ATGTGCGGAGGAGAAGAAGAAAGT |  |
| HGG1 3R | GAGCTTGATGAAGAAACCGATGAAA |  |
| HGG2 5F | GAGGAGCTCGCTGCTTTGTCTT | For *FgHGG2* deletion |
| HGG2 5N | CGGAGAGAGCAGCAACAAGAGAAT |  |
| HGG2 5R | gcacaggtacacttgtttagagGTTGAGAATGACAGGGGGTTAGAGT |  |
| HGG2 3F | ccttcaatatcatcttctgtcgCTTGTGCAGGATATTCTGCTCGTAT |  |
| HGG2 3N | TCATCGCCTCTCAAACTACAAGACA |  |
| HGG2 3R | CAGCCTCCTCTAATCTTCCAACCT |  |
| HGG3 5F | CGATGCCACATCTCTACTCTCACG | For *FgHGG3* deletion |
| HGG3 5N | CCTGGACTACGTTTCGAGAATCACT |  |
| HGG3 5R | gcacaggtacacttgtttagagGCACTAGGGTGTACAGGACGAAAC |  |
| HGG3 3F | ccttcaatatcatcttctgtcgCAAAAAGAGCAAAAAGACGACCC |  |
| HGG3 3N | GCTGGTTTGCTTTTCGGTGTC |  |
| HGG3 3R | CAATGGGCGCTCTTTTGTCTCTA |  |
| HGG4 5F | TCATTGATGAAGTCGATGCGTAAGA | For *FgHGG4* deletion using the CRISPR-Cas9 system |
| HGG4 5N | TCGTCGGAAACGTCACATACATCT |  |
| HGG4 5R-RNP | gcacaggtacacttgtttagagCTCCAATGACGACATTACGCTTT |  |
| HGG4 3F-RNP | ccttcaatatcatcttctgtcgGGCTGGTTATAACACCCTTCAGTCTT |  |
| HGG4 3N | GTCAAGTTGCACGCATGAATCAGT |  |
| HGG4 3R | AAGAGCGTGAGGAGGCACTTACA |  |
| HGG5 5F | TGCAATCGTCTTGACACCATCAT | For *FgHGG5* deletion |
| HGG5 5N | TTCGCAGTTTTCCATCTGTCCAG |  |
| HGG5 5R | gcacaggtacacttgtttagagGCACAGGTGTGAACGCAATAATAG |  |
| HGG5 3F | ccttcaatatcatcttctgtcgTACCCTCCGACTAATCCCTGTCTA |  |
| HGG5 3N | ACGACGGTGTCTACGGAAACTTCT |  |
| HGG5 3R | CCGGCCTTTCTATGGTAAGCAATA |  |
| HGG6 5F | CCTATCCTCCAAGTCTTGCCACA | For *FgHGG6* deletion |
| HGG6 5N | CTCTTTTGGCGGTTGTGTCAGTC |  |
| HGG6 5R | gcacaggtacacttgtttagagGGTGTTGGCTTGTTGGCTCTTA |  |
| HGG6 3F | ccttcaatatcatcttctgtcgGTTTTCTCAGTCGCACAACTTACCT |  |
| HGG6 3N | ATCCCAGGCCAGCTTCAGTATTC |  |
| HGG6 3R | GTACCCCTGTAGCCCACCTTTTC |  |
| HGG7 5F | CTTCTGGGGCACCATATGAATCA | For *FgHGG7* deletion |
| HGG7 5N | TCCGTCTTTGTAAATGGCTGTCC |  |
| HGG7 5R | gcacaggtacacttgtttagagGAGGAGTGAAGCTGAATGGTGAGT |  |
| HGG7 3F | ccttcaatatcatcttctgtcgAATAAAAGTGGTTTGATTTGGCGT |  |
| HGG7 3N | GAGGGTACGTTCATGGCTTGGTTA |  |
| HGG7 3R | GAATGACTCTTCGGCCAGTTTTTC |  |
| HGG8 5F | CATTCACTTCCGACTCCACTACGA | For *FgHGG8* deletion |
| HGG8 5N | AAGAACTCTGAGAAGCGAAAGCGT |  |
| HGG8 5R | gcacaggtacacttgtttagagGCTTTGCGCGATAGGTTAGAATAG |  |
| HGG8 3F | ccttcaatatcatcttctgtcgGGTGAACAGATGAAGCTTGAGTGA |  |
| HGG8 3N | TTCATGGCATTCTGCTTCTGGAC |  |
| HGG8 3R | ACCCAATTTCCCGTCGATAACTC |  |
| HGG9 5F | AATGACTTCTCAGCCCAGCATCA | For *FgHGG9* deletion |
| HGG9 5N | GTCTGTTCCTCGAGCCTGTTGACT |  |
| HGG9 5R | gcacaggtacacttgtttagagGAAATGGTTGCGCTGTGTAAAAT |  |
| HGG9 3F | ccttcaatatcatcttctgtcgTAGTACAAGCCGTCCAATGACAGA |  |
| HGG9 3N | TCCATCTCCGTTCCTGTCTTCTTC |  |
| HGG9 3R | CCGAATCCATCACATATCAGTTGC |  |
| HGG10 5F | TCACAACCCCACTCACATCGTCTA | For *FgHGG10* deletion |
| HGG10 5N | AGCTTTTGTGGCTAACCAACGAGT |  |
| HGG10 5R | gcacaggtacacttgtttagagAATCCCTTGCCACCAAATAAAAA |  |
| HGG10 3F | ccttcaatatcatcttctgtcgTGCAAGGCGTTGTTATGGATTAG |  |
| HGG10 3N | TTGATGGCCTGGATTGTCATTGT |  |
| HGG10 3R | GAAATTCAGTGTGGCATGCTCATC |  |
| HGG11 5F | TGGTATGGGCCTGTCTGTGTCTC | For *FgHGG11* deletion |
| HGG11 5N | GTGACGAGGCCATCTACAAGACG |  |
| HGG11 5R | gcacaggtacacttgtttagagTTTAAAGAATATCGATTGCCGCTC |  |
| HGG11 3F | ccttcaatatcatcttctgtcgATTATGTTTAAAAGCGAGGCGTTC |  |
| HGG11 3N | TTGGTAATTGGTGTTCGTGGTTGT |  |
| HGG11 3R | AGCATCTGATCGCTGGAAGAATG |  |
| HGG12 5F | GCTTGACGTCTCTTTGATGCTTCA | For *FgHGG12* deletion |
| HGG12 5N | ACTGATTGTGGCTGTCTTTCCGA |  |
| HGG12 5R | gcacaggtacacttgtttagagAGGCAAAAGATGAACGAGATGGTA |  |
| HGG12 3F | ccttcaatatcatcttctgtcgCGCGGATTCACTTAGCTATACCA |  |
| HGG12 3N | AAACTACAAGCTCGAGCAGAAGGC |  |
| HGG12 3R | CAGTGTTTTCGACCAGCGTGTG |  |
| HGG13 5F | TCACACCCTGATATTGGTCACGAA | For *FgHGG13* deletion |
| HGG13 5N | TTTTTTTTTCTTGTGTCGCAGGGT |  |
| HGG13 5R | gcacaggtacacttgtttagagTCAAGATTTCTCCGAAGCAACAC |  |
| HGG13 3F | ccttcaatatcatcttctgtcgTCTGTTGGCTTTCAGGGGTGTA |  |
| HGG13 3N | CTTACTCCGACGAGGTTCTGGGTA |  |
| HGG13 3R | AACTTCCGCACCGTTATCCATCT |  |
| HGG14 5F | GATGAGGAGGGCGTCAAGGTAAG | For *FgHGG14* deletion |
| HGG14 5N | AAGAATTGGACGTTTCACCGAGC |  |
| HGG14 5R | gcacaggtacacttgtttagagGTCTTGGAAGGAAGGAAGAAGGAG |  |
| HGG14 3F | ccttcaatatcatcttctgtcgGGCGTCCGTTTTTTACTCATCA |  |
| HGG14 3N | AGCAACAAACGCCACAACAGAAG |  |
| HGG14 3R | CACATACACGTCCGTGTCTGGTTT |  |
| HGG15 5F | CTTTCTCTCCTTTCCCTTCCTGCT | For *FgHGG15* deletion |
| HGG15 5N | ACAATAGATTTGGGGTCCGCATAG |  |
| HGG15 5R | gcacaggtacacttgtttagagAGCAGTGCAGTGGTTTTTCTTCTT |  |
| HGG15 3F | ccttcaatatcatcttctgtcgATGGGAGGCAGCGATGACTTA |  |
| HGG15 3N | TCGCCTTGAAGGGATGGAACTAC |  |
| HGG15 3R | TTGGGAGAGGTAACCTGGATTGC |  |
| HGG16 5F | ATTGGATTTCTACATCGTCGGGAA | For *FgHGG16* deletion |
| HGG16 5N | ACCTCCTGGTCCAACTCGTAGAGA |  |
| HGG16 5R | gcacaggtacacttgtttagagCGGATGTCACTTTTTGGATGGA |  |
| HGG16 3F | ccttcaatatcatcttctgtcgTGCATTGGCGTTTATGTATTGATT |  |
| HGG16 3N | CCTTGGCCTTTTGTTGCTGTTCT |  |
| HGG16 3R | TCAATGACTCTCGCTGTAACTCGG |  |
| HGL1 5F | GGAAACTTGTGATACTTGCCCGTT | For *FgHGL1* deletion |
| HGL1 5N | TCATTAGAGCACACGAGCAGAGACA |  |
| HGL1 5R | gcacaggtacacttgtttagagTGGTAATAATTGCGAAGGGAGC |  |
| HGL1 3F | ccttcaatatcatcttctgtcgGCCGGAGTCTAGAGCTCATTTACA |  |
| HGL1 3N | TGAATCCTCAAGTCGAATGTCACG |  |
| HGL1 3R | TGGGTTGGCACTATGGATTTCAG |  |
| HGL2 5F | GCAAGACTCAACTGCCTGACTGAC | For *FgHGL2* deletion |
| HGL2 5N | TCGGATTGACCAGTTTTGTGTGAC |  |
| HGL2 5R | gcacaggtacacttgtttagagAACTCCCAATGAGACTCAAGATGC |  |
| HGL2 3F | ccttcaatatcatcttctgtcgTGGCGTTTGAAGTAGCTGAACAT |  |
| HGL2 3N | ATGCAACGTGAGAGTCCAGTCAGA |  |
| HGL2 3R | AAATTCTGAGGAAACTCACGGACG |  |
| HGL3 5F | GTACATGATCTAGACTCGGGCGTG | For *FgHGL3* deletion |
| HGL3 5N | CAATGCCTGCTCTCGGTGTTATC |  |
| HGL3 5R | gcacaggtacacttgtttagagTTGGTAGCAGTTGAGTAAGCATTCC |  |
| HGL3 3F | ccttcaatatcatcttctgtcgATTCGACGGACGTTGCATTTATA |  |
| HGL3 3N | AGTGGCGACAACGTTTTCTCATAA |  |
| HGL3 3R | CTTTGAGGGTAGGGTCTTGCTTCA |  |
| HGL4 5F | GTGAAAAGATGGGCGAAAGGAGT | For *FgHGL4* deletion |
| HGL4 5N | CCTGAAGGGTGTGCTGAGTCAAC |  |
| HGL4 5R | gcacaggtacacttgtttagagTTGTCTATTGTGCCTGTGGTGAAC |  |
| HGL4 3F | ccttcaatatcatcttctgtcgATAGCCACGAGCAAAAGTAACGAG |  |
| HGL4 3N | TGCTGTTGCTGTTGGTTCTTCCTA |  |
| HGL4 3R | TGGTGGTTGTTCCCAAAAGACAG |  |
| HGL5 5F | CTCAATTCAAAATGGCACAAAACG | For *FgHGL5* deletion |
| HGL5 5N | TGGCTGGAATGTGGTATGTCTCAT |  |
| HGL5 5R | gcacaggtacacttgtttagagTTAACTGCGGGGAGATTGTAACC |  |
| HGL5 3F | ccttcaatatcatcttctgtcgCACGTTTAACTCATATGAGGTGCG |  |
| HGL5 3N | AGCGCTCAATCGGATGAACTATTC |  |
| HGL5 3R | TCTCAAAGTCAGACATCGCCAGAA |  |
| HGL6 5F | AGGGATACGGCCACGTCAACTAT | For *FgHGL6* deletion |
| HGL6 5N | CTGCGGCGATGAATAGACACTCT |  |
| HGL6 5R | gcacaggtacacttgtttagagTTCAATTCACCGCATCTTGTAAAG |  |
| HGL6 3F | ccttcaatatcatcttctgtcgCTGTCAGCGATCGTGATTCTGTC |  |
| HGL6 3N | ATAGCTGCGGTTCATTCACATCG |  |
| HGL6 3R | ATACATGGCCTCGCAAGCATAATA |  |
| HGP1 5F | AGTGTCTGCCCCGGAATAGTCTTA | For *FgHGP1* deletion |
| HGP1 5N | TCCAATTCTCAGCAACCAACGAG |  |
| HGP1 5R | gcacaggtacacttgtttagagTGTCTGTCGCAAGTTGGAGAAGTA |  |
| HGP1 3F | ccttcaatatcatcttctgtcgAGCACAGTATGGAAAGTAGACGGG |  |
| HGP1 3N | CGCATTACCCCTGAGATCGTCTAC |  |
| HGP1 3R | CGATGGAAAGACCAAGGAGAACAT |  |
| HGP2 5F | CGATGAGAATGAGCGTGGAGTAGA | For *FgHGP2* deletion |
| HGP2 5N | ACTTCCCTCTTTTCTCAACGCCTC |  |
| HGP2 5R | gcacaggtacacttgtttagagGAATGAGGAGGTGGAGGTGAAAC |  |
| HGP2 3F | ccttcaatatcatcttctgtcgCGTCTTCTGTACCCTAGTTCCATTG |  |
| HGP2 3N | CAACACGATCACCGGACTATCTCA |  |
| HGP2 3R | CTGGAGAGGCCAACTTTTCTGCTA |  |
| HGP4 5F | AAACCGTGTTTGATGGGAATGAAC | For *FgHGP4* deletion |
| HGP4 5N | TGATGAAGCACCACAATGAAACCT |  |
| HGP4 5R | gcacaggtacacttgtttagagTGAGTGAAGGGGTATCGAAGAAAA |  |
| HGP4 3F | ccttcaatatcatcttctgtcgGTTCGGACTGTTATTTGGGATGAT |  |
| HGP4 3N | CTACGAGGGCTCTTTGTCCGATT |  |
| HGP4 3R | CGACTCTTTCTTCAAGCCCGACTA |  |
| HGP5 5F | ATCGTCAACAAAAAAAAAGCCAGC | For *FgHGP5* deletion |
| HGP5 5N | TTGATGGCAAAGGCTTGAAACTT |  |
| HGP5 5R | gcacaggtacacttgtttagagAGGTGGAAGGAAGACGAGTTACG |  |
| HGP5 3F | ccttcaatatcatcttctgtcgGGCATAGGTGGTGGATGAAAAATA |  |
| HGP5 3N | CCTTGGCATTCTCTGAAGTCTTGG |  |
| HGP5 3R | CGGATGTTTGATGCTGAAGTGAGT |  |
| HGP7 5F | GGCCTCGACCCTATTCAGTAAATG | For *FgHGP7* deletion |
| HGP7 5N | GTGTGGCACTGTACTGCACCGTAT |  |
| HGP7 5R | gcacaggtacacttgtttagagGTCCGAGTGAATAAAGATGGTTGG |  |
| HGP7 3F | ccttcaatatcatcttctgtcgGATTTACGGCTTGAATTTTTGGTG |  |
| HGP7 3N | ATCATCACCCATCCTCTCGTCCTA |  |
| HGP7 3R | CTCAATGTTGTGGACAAGAGTCGC |  |
| HGP8 5F | CTTCAGCACCATCATCCTCTCAAA | For *FgHGP8* deletion |
| HGP8 5N | GCTGGAAACACAAGGGACAAAAGT |  |
| HGP8 5R | gcacaggtacacttgtttagagATCGATGATGATGGCTTCCTATTC |  |
| HGP8 3F | ccttcaatatcatcttctgtcgGCAAGGGATATAGGGAAAGGAGTC |  |
| HGP8 3N | GAACCCCTCTACGGCTACGATTCT |  |
| HGP8 3R | ACCACGATTGACGCCAATAAAAAT |  |
| GEN/REV | CTCTAAACAAGTGTACCTGTGC | For amplification of the *GEN*-resistant cassette |
| GEN/FOR | CGACAGAAGATGATATTGAAGG |  |
| HGG4 5Rcom | gaacagctcctcgcccttgctcacGAAGTCAAGGCTCTCTTTCTTTGGC | For *FgHGG4* complementation |
| HGG4 3Fcom | cctccactagctccagccaagccCTTGCATCAAGTCTGCCCTTTTAT |  |
| HGG10 5Rcom | gaacagctcctcgcccttgctcacGTGAGAAATATACTGGAACACGGCAG | For *FgHGG10* complementation |
| HGG10 3Fcom | cctccactagctccagccaagccTGCAAGGCGTTGTTATGGATTAG |  |
| HGG13 5Rcom | gaacagctcctcgcccttgctcacCGCCATCTTCTGCCTCTTCG | For *FgHGG13* complementation |
| HGG13 3Fcom | cctccactagctccagccaagccTCTGTTGGCTTTCAGGGGTGTA |  |
| GFP-F1 | GTGAGCAAGGGCGAGGAGCTG | For amplification of the *GFP*-*HYG* resistant gene cassette |
| HYG-F1 | GGCTTGGCTGGAGCTAGTGGAGG |  |
| HGG10 HYG 5R | CGACCGGGAACCAGTTAACAAAATCCCTTGCCACCAAATAAAAA | For *FgHGG10* deletion in Δ*fghgg4* mutant |
| HGG10 HYG 3F | CCTCCACTAGCTCCAGCCAAGCCTGCAAGGCGTTGTTATGGATTAG |  |
| HGG13 HYG 5R | CGACCGGGAACCAGTTAACAATCAAGATTTCTCCGAAGCAACAC | For *FgHGG13* deletion in Δ*fghgg4* and Δ*fghgg10* mutants |
| HGG13 HYG 3F | CCTCCACTAGCTCCAGCCAAGCCTCTGTTGGCTTTCAGGGGTGTA |  |
| HYG-R | TTGTTAACTGGTTCCCGGTCG | For amplification of the *HYG* resistant gene cassette |
| HYG-F1 | GGCTTGGCTGGAGCTAGTGGAGG |  |
| HGL7 P-5F | GGTCATGGCTATGCAGTCACAGTCT | For construction of *P_ZEAR_-HGL7* |
| HGL7 P-5N | ATACGACCGTAACGCCTTCTTCAT |  |
| HGL7 P-5R | tccactagctccagccaagccAAGAAACTCGTCGATGAACTCTGG |  |
| HGL7 P-3F | gagagaacgaaagtaaccatgATGGCTGCCAGACAGCCAGCCCGG |  |
| HGL7 P-3N | GCCACCGGTGTCCCTATCTATG |  |
| HGL7 P-3R | TGTGATGGCCGAAATGGAACA |  |
| HGP3 P-5F | CCAATCGTCCAAACAGCACAATAC | For construction of *P_ZEAR_-HGP3* |
| HGP3 P-5N | CAAACCAACCTCACAACAACATAAAA |  |
| HGP3 P-5R | tccactagctccagccaagccGTTTAACACTTTTTTGTAGGCGTGC |  |
| HGP3 P-3F | gagagaacgaaagtaaccatgATGCGTCCCGAAGTCGAGCAGG |  |
| HGP3 P-3N | CGGTGGGGCAGTTATCAATGTG |  |
| HGP3 P-3R | AAGCAGTTCAGGTACACGCCAGTA |  |
| HGP6 P-5F | CTGGGAGTTGCCTTGTTTGTCAC | For construction of *P_ZEAR_-HGP6* |
| HGP6 P-5N | GGGAAGGGGTTTGTGTTGTAGTTG |  |
| HGP6 P-5R | tccactagctccagccaagccAAGAAACACAGCAGAAGCAAAGGA |  |
| HGP6 P-3F | gagagaacgaaagtaaccatgATGGCCTTCAACTTCAACTGG |  |
| HGP6 P-3N | ACCGGCTTCATCTTCTGTTTCTGT |  |
| HGP6 P-3R | CTTCTTGCGTCCTGCTCGTGA |  |
| zear-r2 | CATGGTTACTTTCGTTCTCTCTGGTC | For amplification of the promoter region of *ZEAR* gene with Hyg-F1 primer |
| TRX qrtF | TTCACCACATTACTTCAAACGACGAG | For real time-PCR of *TRX* |
| TRX qrtR | TGTGCTTAGTGGAGAGTTGCTCGTAG |  |
| CYP1 qrtF | TCAAGCTCAAGCACACCAAGAAGG | For real time-PCR of *CYP1* |
| CYP1 qrtR | GGTCCGCCGCTCCAGTCT |  |
